# Metabolomic profiling and its association with the bio-efficacy of *Aspergillus niger* strain against *Fusarium* wilt of guava

**DOI:** 10.3389/fmicb.2023.1142144

**Published:** 2023-04-24

**Authors:** R. Gangaraj, Aditi Kundu, Virendra Singh Rana, Amrita Das, Gautham Chawla, G. Prakash, Rubin Debbarma, A. Nagaraja, Naresh Kumar Bainsla, Navin Chandra Gupta, Deeba Kamil

**Affiliations:** ^1^Division of Plant Pathology, ICAR-Indian Agricultural Research Institute, New Delhi, India; ^2^Division of Agricultural Chemicals, ICAR-Indian Agricultural Research Institute, New Delhi, India; ^3^Division of Nematology, ICAR-Indian Agricultural Research Institute, New Delhi, India; ^4^Division of Fruits and Horticultural Technology, ICAR-Indian Agricultural Research Institute, New Delhi, India; ^5^Division of Genetics, ICAR-Indian Agricultural Research Institute, New Delhi, India; ^6^ICAR-National Institute for Plant Biotechnology, New Delhi, India

**Keywords:** bio-control, *Aspergillus niger*, internal transcribed spacer, *Fusarium oxysporum* f. sp. *psidii*, gas chromatography-mass spectrometry, secondary metabolites

## Abstract

Bio-control agents are the best alternative to chemicals for the successful management of plant diseases. The fungus *Aspergillus niger* is known to produce diverse metabolites with antifungal activity, attracting researchers to exploit it as a bio-control agent for plant disease control. In the present study, 11 *A. niger* strains were isolated and screened for their antagonism against the guava wilt pathogen under *in vitro* and *in planta* conditions. Strains were identified morphologically and molecularly by sequencing the internal transcribed spacer (ITS), β-tubulin, and calmodulin genes. The strains were evaluated through dual culture, volatile, and non-volatile methods under an *in vitro* study. AN-11, AN-6, and AN-2 inhibited the test pathogen *Fusarium oxysporum* f. sp. *psidii* (FOP) at 67.16%, 64.01%, and 60.48%, respectively. An *in planta* study was conducted under greenhouse conditions with 6 months old air-layered guava plants (var. Allahabad Safeda) by pre- and post-inoculation of FOP. The AN-11 strain was found to be effective under both pre- and post-inoculation trials. Furthermore, gas chromatography–mass spectrometry (GC–MS) analysis was carried out to characterize the volatile compounds of the most potential strain, *A. niger*. The hexane soluble fraction showed the appearance of characteristic peaks of hexadecenoic acid methyl ester (4.41%), 10-octadecanoic acid methyl ester (3.79%), dodecane (3.21%), undecane (3.19%), gibepyrone A (0.15%), 3-methylundecane (0.36%), and citroflex A (0.38%). The ethyl acetate fraction of the bio-control fungi revealed the occurrence of major antifungal compounds, such as acetic acid ethyl ester (17.32%), benzopyron-4-ol (12.17%), 1,2,6-hexanetriol (7.16%), 2-propenoic acid ethanediyl ester (2.95%), 1-(3-ethyloxiranyl)-ethenone (0.98%), 6-acetyl-8-methoxy dimethyl chromene (0.96%), 4-hexyl-2,5-dihydro dioxo furan acetic acid (0.19%), and octadecanoic acid (1.11%). Furthermore, bio-control abilities could be due to hyper-parasitism, the production of secondary metabolites, and competition for sites and nutrients. Indeed, the results will enrich the existing knowledge of metabolomic information and support perspectives on the bio-control mechanism of *A. niger*.

## 1. Introduction

Guava (*Psidium guajava* L.), an important fruit extensively grown in tropical and subtropical countries throughout the world, belongs to the family Myrtaceae. It is rich in vitamin C, phosphorus, iron, and calcium and has anti-oxidant properties (Kumari and Choudhary, 2019; Kumar et al., 2021a). In India, it is cultivated in various states (Bihar, Uttar Pradesh, Maharashtra, Andhra Pradesh, Gujarat, Karnataka, Orissa, Tamil Nadu, and Chhattisgarh), with diverse varieties of cultivars. Wilt is an important disease threatening guava production worldwide, leading to significant yield loss, and is considered a national constraint in India. The disease was first reported in Taiwan in 1926. It was initially recorded in India in Allahabad, Uttar Pradesh (Das Gupta and Rai, 1947). Disease prevalence ranges from 75% to 90%, while disease severity ranges from 30% to 55%. The disease is most prevalent in the states of Uttar Pradesh, Uttarakhand, West Bengal, Bihar, Rajasthan, Punjab, and Madhya Pradesh, causing a 30% yield loss. The infected trees initially show yellowing of the older leaves with slight curling at the terminal ends, and then, they turn reddish and start shedding leaves. The twigs become bare and do not form new leaves or flowers. The fruits remain underdeveloped, black, and hard. The presence of pathogen propagules in the xylem vessel disrupts the flow of water, resulting in the complete wilting of plants. The severity is worse in older trees than in young trees. Under severe conditions, the complete death of the plants results in 100% yield loss (Gupta et al., 2010; Suresh et al., 2019; Singh et al., 2021). Significant economic losses are also recorded in Florida (U.S.A.), Cuba, Brazil, Bangladesh, Pakistan, South Africa, Taiwan, and Australia (Junqueira et al., 2001; Lim and Manicom, 2003; Schoeman, 2011; Hussain et al., 2014; Shah et al., 2019).

The disease is reported to be associated with several pathogenic fungi. However, Fusarium spp. is considered an important pathogen characterized worldwide. Among them, *Fusarium oxysporum* f. sp. *psidii* (FOP) is predominant, and its pathogenicity is also confirmed under field conditions (Gupta et al., 2010; Gangaraj et al., 2022). Since it is a soil-borne disease, once established in the field, it is very difficult to control. Several fungicides have been used to control the guava wilt disease, but the management is not successful. The chemicals have been reported to increase the aggressiveness of the pathogen by increasing spore production (Misra and Pandey, 1999). The use of potential bio-control agents (BCAs) is an alternative, broad-spectrum, eco-friendly, and economical strategy to combat plant diseases. The BCAs remain viable for a long time in the soil, providing substantial and consistent disease control. In addition to controlling the pathogen, they also promote the growth and vigor of the plant. BCAs, such as *Trichoderma harzianum, T. asperellum, Aspergillus* spp., *Gliocladium virens*, and *Penicillium* spp., have been reported to be efficient for the management of wilt disease caused by *Fusarium* spp. *Aspergillus niger* was found to be promising in controlling the guava wilt disease caused by *F. oxysporum* f. sp. *psidii* and *F. solani* under both laboratory and field conditions (Singh et al., 2003; Gupta and Misra, 2009; Sharma et al., 2011).

*Aspergillus niger* is a filamentous ascomycetous soil-invading fungus that is effective against most soil-borne pathogens causing wilts (Mandol, 1998; Mukherjee and Sen, 1998; Gupta and Misra, 2009; Nayak and Vibha, 2017). The genus *Aspergillus* was initially illustrated by Micheli (1729) and later by Link (1809). It is considered generally recognized as safe (GRAS) by the Food and Drug Administration (Perrone et al., 2012). A wide range of extracellular enzymes and secondary metabolites are produced by *A. niger*, which has antimicrobial activity against *F. oxysporum, F. solani, Pythium* spp., *Sclerotinia sclerotiorum*, and *Pyricularia oryzae*. These include pyranones, alkaloids, cyclopentapeptides, polyketides, and sterols (Mondal et al., 2000; Patibanda and Sen, 2005; Idan et al., 2017; Yu et al., 2021). It is also reported to improve plant growth through the production of growth-promoting substances (Nielsen et al., 2009). *A. niger* produces a number of bioactive secondary metabolites with antimicrobial activity. Even though *A. niger* is found to be a promising bio-control agent, very few formulations, *viz*. Kali Sena (AN-27) and Pusa Mrida (AN-17), are available for commercial use to control phytopathogens (Mondal et al., 2000; Singh et al., 2003).

Moreover, there is a lack of availability of suitable bio-control strains of *A. niger*, and only a few commercial products are available in India. There is a lack of reports available on the secondary metabolite profiling of *A. niger* during interaction with the pathogen. The characterization of volatile organic compounds (VOCs) in GC–MS is well known because of its high sensitivity and high separation capability. In recent times, VOC-based formulations in the field have gained scope in the field of crop protection against plant pathogens (Korpi et al., 2009; Darshan et al., 2021). There is a need for a detailed understanding of the role of volatile metabolites to improve biological control efficacy and to explore and understand mechanisms and metabolites produced to improve the bio-efficacy of *A. niger* against pathogens associated with wilt disease in Indian conditions. Therefore, the present study was carried out to evaluate *A. niger* strains antagonistic activity against the guava wilt pathogen under *in vitro* and *in planta* conditions and to characterize the bioactive compounds produced during the interaction between the bio-control agent and the test pathogen. This information will facilitate extensive applications of formulation in the field of bio-control in the future for the successful management of guava wilt disease.

## 2. Materials and methods

### 2.1. Collection and isolation of the fungus

The soil and infected root samples showing symptoms of rotting at the basal region, discoloration of cortical tissues, and detachment of bark from the cortex were collected from a guava field at the ICAR—Indian Agricultural Research Institute (IARI), New Delhi. Furthermore, root samples were washed under running tap water for 2 min and cut into small pieces (<2 cm). Root pieces were surface sterilized for 1 min with sodium hypochlorite (1%) and then rinsed one more time with sterile double-distilled water (Suresh et al., 2019). The sterilized root pieces (<2 cm) were placed on potato dextrose agar (PDA) Petri plates and incubated for 7 days at 26 ± 1°C. Through the hyphal tip method, pure cultures of different isolates of the pathogens were obtained by culturing on a water agar medium, sub-culturing, and maintaining PDA slants throughout the study. Based on morphological and molecular characteristics the isolate was confirmed as *F. oxysporum* f. sp. *psidii*. A pot experiment was conducted to confirm the pathogenicity of the guava wilt pathogen using 6 months old, air-layered guava plants (variety Allahabad Safeda) following the standard method described by Misra and Pandey (2000). The stem hole technique was performed; the wound was made with the help of a sterile blade, and a 10-day-old culture of *F. oxysporum* f. sp. *psidii* grown on PDA media with a spore concentration of 10^7^ conidia/ml was inoculated at the wound, which was wrapped with paraffin to keep away from other contaminants. The symptoms of yellowing and shredding of the leaves, followed by curling of terminal branches, were observed after 15 days of inoculation. At the end of 4 months of inoculation, complete wilting was recorded and the cross-section showed brownish discoloration of the vascular system upon splitting. The pathogen was re-isolated from plants showing disease symptoms as per the methodology described (Suresh et al., 2019). *Fusarium oxysporum* f. sp. *psidii* strain (ITCC-8288) was deposited in the Indian Type Culture Collection (ITCC), New Delhi, and NCBI GenBank accession numbers (MN972593) were obtained (Gangaraj et al., 2022). In total, 11 strains of *A. niger* were collected, and isolated and pure cultures were obtained through the hyphal tip method by culturing on water agar medium, sub-cultured, and maintained on PDA slants at 26 ± 1°C (Table 1). Furthermore, all the strains were studied for their morphological characteristics (Diba et al., 2007; Javadi et al., 2012).

**Table 1:** Microscopic characteristics used for the identification of eleven strains of *A. niger* grown on potato dextrose agar (PDA) at 7 days after inoculation (DAI).

**Strain**	**Location**	**Source**	**ITS^a^**	**β-tubulin^a^**	**Calmodulin^a^**	**Colony color**	**Conidia characteristics**		
							**Shape and surface morphology**	**Size (**μ**m)**	**Color**
AN-1	New Delhi	Soil	MW692855	OQ507885	OQ507894	White	Sub-globose, slightly rough, and irregular	3.87	Black
AN-2	New Delhi	Soil	MW692856	OQ507886	OQ507873	White	Globose, very rough, and irregular	4.38	Black
AN-3	New Delhi	Soil	MW692857	OQ507890	OQ507893	Milky white	Globose, rough, and irregular	3.46	Black
AN-4	New Delhi	Soil	MW692858	OQ507887	OQ507898	White	Globose, rough, and irregular	3.98	Cream
AN-5	Chandigarh	Waste of sugarcane industries	MW692859	OQ507888	OQ507877	White	Sub-globose and rough (ornamented with stripes)	4.49	Black
AN-6	Kanpur	Soil	MW692860	OQ507889	OQ507892	Creamy white	Sub-globose, rough, and irregular	4.16	Black
AN-7	Bikaner	Coconut rhizosphere	MW692861	OQ507883	OQ507896	White	Globose and slightly rough	4.02	Dark brown
AN-8	Hisar	Degrading wood	MW692862	OQ507884	OQ507874	White	Globose, rough, and irregular	4.63	Dark brown
AN-9	New Delhi	Soil, IARI	MW692863	OQ507891	OQ507895	Buff	Globose, smooth, and ornamented	3.68	Dark brown
AN-10	New Delhi	Soil, IARI	MW692864	OQ507882	OQ507875	White	Globose, slightly rough, and irregular	3.56	Black
AN-11	New Delhi	Soil, IARI	MW692865	OQ507881	OQ507876	Milky white	Sub-globose, rough, and irregular	3.94	Dark brown

a GenBank accession number.

### 2.2. Genomic DNA isolation and phylogenetic analysis

Genomic DNA extraction from 11 *A. niger* strains was performed using a modified CTAB (cetyl trimethyl ammonium bromide) procedure (Cullings, 1992). PCR was performed using amplification of the ITS, β-tubulin, and calmodulin regions. The amplification of the ITS region was carried out using universal primers ITS1 5′-TCCGTAGGTGAACCTGCGG-3′ and ITS4 5′-TCCTCCGCTTATTGATATGC-3′ (White et al., 1990). Bt2a 5′-GGTAACCAAATCGGTGCTGCTTTC-3′ and Bt2b 5′-ACCCTCAGTGTAGTGACCCTTGGC-3′ primers were used for the amplification of β-tubulin region (Nasri et al., 2015). Similarly, primers CAL-228F 5′-GAGTTCAAGGAGGCCTTCTCCC-3′ and CAL-737R 5′-CATCTTTCTGGCCATCATGG-3 were used for the amplification of the calmodulin region (Carbone and Kohn, 1999). A total volume of 25 μl of PCR mixture consists of 12.5 μl of DreamTaq Green PCR Master Mix (Thermo Scientific, India) (containing 0.25 mM of each dNTP, 2 mM MgCl_2_, and Taq DNA polymerase), 9.5 μl of nuclease-free water, 1 μl of primer (10 pmol/μl of each forward and reverse primer), and the remaining 1 μl (100 ng/μL) of DNA template was used for amplification. PCR was performed with initial denaturation for 5 min at 94°C, 35 cycles of denaturation at 94°C for 1 min, annealing temperature at 56°C for 1 min, primer extension at 72°C for 2 min, and final primer extension at 72°C for 5 min for the ITS and β-tubulin regions. The PCR for the calmodulin region was performed with an initial denaturation temperature of 94°C for 5 min, 35 cycles of denaturation at 94°C for 1 min, annealing temperature at 52°C for 1 min, primer extension at 72°C for 2 min, and a final primer extension at 72°C for 5 min. The electrophoresis was performed using 1.2% agarose gel with 0.5 mg/μl ethidium bromide in the 1 × TAE buffer (100 V, 400 mA for 35 min) to visualize the PCR product. The 1 Kb DNA Marker was used to estimate the size of the PCR product (Thermo Fisher Scientific, USA), which was further purified (Gene JET^TM^, Thermo Fisher Scientific, USA) and sequenced (Bioengineering, India).

For the phylogenetic analysis, three gene sequences (ITS + β-tubulin + calmodulin) were used. NCBI nucleotide BLAST analysis was performed to compare the retrieved nucleotide sequences with those in the GenBank database. The sequences were constructed, modified, and aligned using Clustal W in the BioEdit sequence alignment editor. The sequences were constructed, modified, and aligned using BioEdit, which was used to perform the cluster analysis. The ambiguously aligned sites were eliminated, and gaps were treated as missing data. Furthermore, the sequences were artificially aligned, and ambiguous areas caused by insertions and deletions (indel) were removed. The sequences were concatenated after alignment, and MEGA version 7 was used largely for “Maximum Likelihood” tree construction (Tamura and Nei, 1993; Kumar et al., 2016). The ex-type strain of *A. niger* NRRL 326 (accession numbers: EF661186, EF661089, and EF661154) was employed as an outgroup, and *A. flavus* strain NRRL 1957 (accession numbers: AF027863, EF661485, and EF661508) was utilized to construct the phylogenetic tree (Samson et al., 2014). To assess the stability of branches, a bootstrap analysis with 1,000 replications was performed.

### 2.3. *In vitro* antagonistic assays

A dual culture assay was performed to test the *in vitro* antagonistic activity of *A. niger* strains against FOP (Chen et al., 2016). *A. niger* strains and FOP were grown on PDA plates for 7 days at 26 ± 1°C. A 5 mm disk of *A. niger* and the test pathogen (FOP) were placed exactly opposite each other at 2 cm from the periphery on the PDA plate and incubated at 26 ± 1°C for 7 days. The Petri plates containing only pathogens (FOP) were maintained separately as controls. The entire experiment was performed with three replications per treatment. The radial growth (mm) and percent inhibition were measured and calculated following the formula given by Garcia (1991): IRG (%) = 100 [(R1 – R2)/R1], where R1 denotes the farthest radial growth of the pathogen in the direction of the antagonist (control) and R2 denotes the distance on a line between the inoculation positions of the antagonist and the pathogen (Morton and Stroube, 1955).

The procedure outlined by Dennis and Webster (1971a) was used to conduct the volatile test. On the PDA plate, a 5 mm mycelial disk of *A. niger w*as placed at the center, and on another plate, a pathogen (FOP) was inoculated in the same manner. The two plates were taped together using paraffin, and they were kept at 26°C for 7 days. The control was maintained without an antagonist and PDA medium in the opposite Petri plate. The non-volatile test was assessed using the process outlined by Dennis and Webster (1971b). Strains of *A. niger* were grown in PDB for 15 days and later filtered through a micropore filter. The final concentration was increased to 10% (v/v) and added to PDA media. A 5 mm disk of FOP was placed after solidification. In addition, control plates were kept without adding the culture filtrate to them. The percent mycelial growth inhibition was calculated after 7 days of inoculation as described in the dual culture assay. The entire experiment was conducted with three replications. Furthermore, based on the *in vitro* bio-efficacy assay, *A. niger* strains were characterized into three clusters, i.e., Group-1 with high potential strains showing 75–100% inhibition, Group-2 with moderate potential strains having 50–75% inhibition, and Group-3 with low potential strains having <50% inhibition of FOP growth.

### 2.4. *In planta* bio-control assay

The *A. niger* strains were evaluated using 6 months old air-layered guava plants (var. Allahabad Safeda) through pre- and post-inoculation treatment under greenhouse conditions against FOP. *A. niger* inoculum was cultured on sorghum grains. Several 500 ml conical flasks containing 250 g of sorghum seeds were sterilized for 2 days. Furthermore, 6-days-old *A. niger* strains growing on PDA were transferred into the sterilized sorghum seeds and incubated for 8–10 days. *A. niger* strains were further mass multiplied in 500 g of sterilized farmyard manure by adding 10 g of sorghum seeds grown in *A. niger* inoculum and incubated for 15 days (Misra and Prasad, 2004; Misra et al., 2004). Moreover, 6 months old air-layered guava plants (var. Allahabad Safeda) were grown in pots (30 cm diameter) for *in planta* evaluation at the Division of Plant Pathology, ICAR-IARI, New Delhi. The potting mixture was made up of soil and compost (1:1) that was sterilized two times. The control was maintained by inoculating only pathogens (FOP), achieving negative control with only distilled water and treating with carbendazim (0.2%). Furthermore, 7 day old FOP culture was used as a pathogen inoculum source. To establish the wilt disease, a 5 mm mycelial plug of the test pathogen was inoculated by making a wound in the stem of the guava plant, followed by covering it with cotton and adhesive tape as described by Misra and Pandey (2000). In addition, 30 ml of PDB containing FOP inoculum was also incorporated into the soil (root zone) for better establishment. In the case of pre-inoculation treatment, the test pathogen was inoculated onto the guava plants, and each *A. niger* strain was then added separately after 15 days, and vice versa in the case of the post-inoculation method. The percent reduction of wilt incidence (healthy, partially wilted, and completely wilted) was recorded as the average of data measured at 7 days intervals for up to 9 months. The percent disease incidence was calculated (Hussain et al., 2014). Using the modified scale, the wilting of guava plants in a field was scored (Misra and Pandey, 2000). The disease severity scale is as follows: 0 = healthy plants; 1 = yellowing of leaves; 2 = yellowing of leaves with 25% wilting of plants; 3 = yellow–brown discoloration with 50% wilting; 4 = pronounced wilting and entire plants started dying (75%); 5 = complete wilting and death of the plant. Each treatment consisted of three replications, and the experiment was repeated two times and was performed in a randomized complete block design (RCBD).

### 2.5. Microscopic study of hyphal interactions between antagonist (*A. niger*) and pathogen (*F. oxysporum* f. sp. psidii)

In order to elucidate the mechanisms of antagonism used by *A. niger* against FOP under *in vitro* conditions, a dual microculture technique was used (Chen et al., 2016). The Petri plates containing pathogens and antagonists were incubated at 26 ± 1°C for 7 days. Fungal colonies were picked from the interaction part using an inoculating needle and placed on the slide which was mounted in 50% (v/v) glycerol. The slides were covered with a cover slip (22 mm × 22 mm), observed, and photo-micrographs were taken using a Cilika BT-P Biological Digital Microscope (MedPrime Technologies Pvt. Ltd. Thane, India).

### 2.6. Scanning electron microscopy (SEM) analysis of dual culture plate

Microscopic image analysis was carried out following the procedure of Darshan et al. (2021). A small piece of mycelium along with some agar was taken from the inhibition zone. Specimens were fixed using chilled (4°C) 2.5% glutaraldehyde (Sigma-Aldrich) prepared in 0.1 M phosphate buffer for 2 h at room temperature. They were rinsed three more times for 15 min at a pH of 7.4 in 0.1 M phosphate buffer (Fisher Scientific). Fixed samples were dehydrated in a graded ethanol series (30%, 50%, 70%, 80%, 95%, and 100% ethanol, 15 min each) after 12 h of refrigeration. For drying the samples, hexamethyldisilazane (Sigma-Aldrich) was utilized. The dried samples were attached to the aluminum specimen mounts using a colloidal silver paste, and then 24 nm of gold palladium was sputtered on top. Samples were evaluated and documented using a scanning electron microscope (SEM) (Zeiss, EVO MA10) at an accelerating voltage of 20 kV/EHT and 10 Pa.

### 2.7. Secondary metabolites profiling

#### 2.7.1. Extraction of bio-control fungi

**Figure 1:**
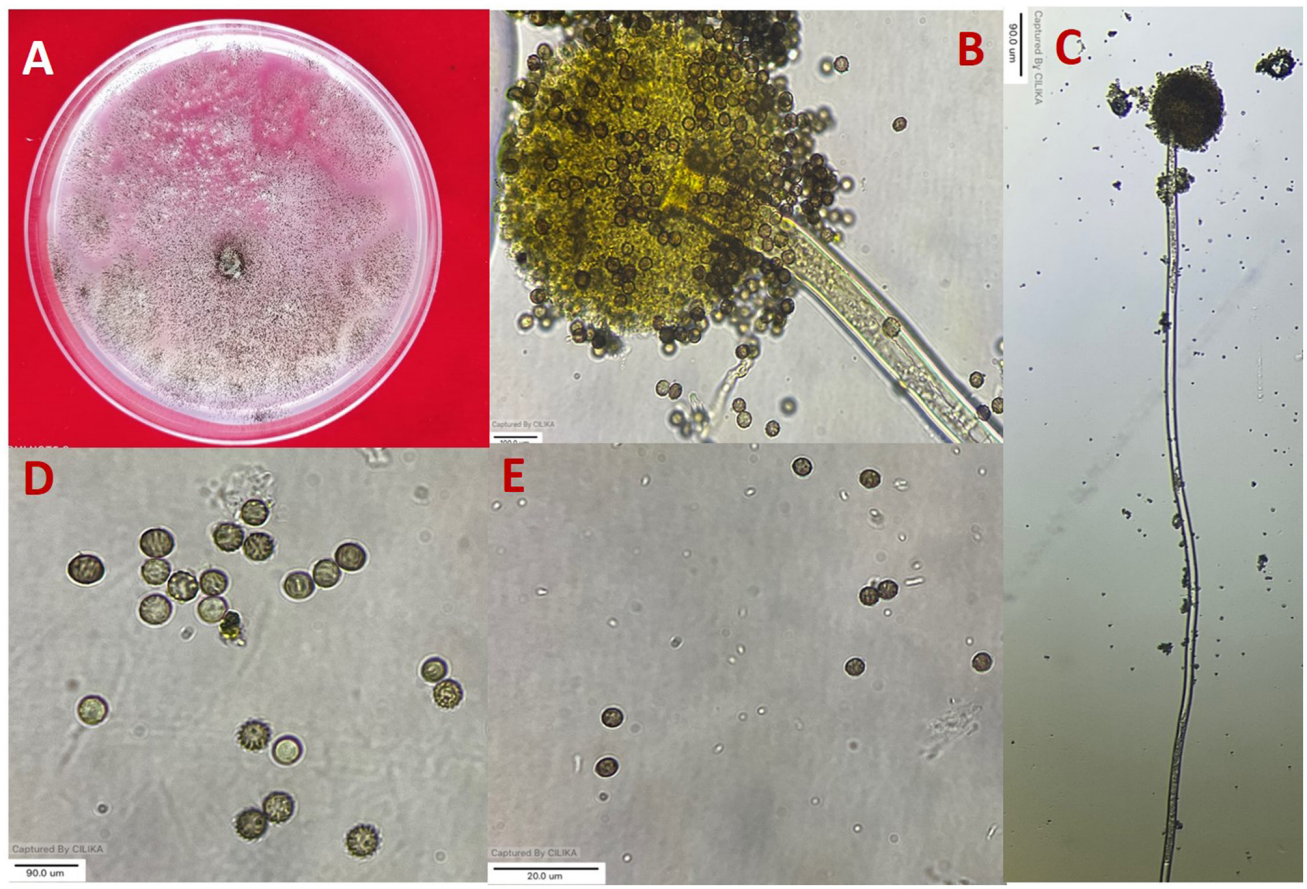
Morphological characterization of *A. niger* **(A)** colony on potato dextrose agar (PDA). **(B, C)** Conidiophore and vesicle-containing conidia. **(D, E)** Conidia.

Following a prior procedure described by Lykholat et al. (2021), with only minor adjustments, the extraction of metabolites was carried out to investigate the antagonistic ability to control *A. niger* (AN). Briefly, a small piece of fungal mycelium (FOP) was transferred to a 500 ml Erlenmeyer flask containing 100 ml of potato dextrose broth (PDB), which had been previously autoclaved at 121°C for 40 min. The medium was cultured for 15 days at 26 ± 1°C with continual 160 rpm shaking in an incubator shaker following inoculation. Mycelial biomass was then separated by filtering it through a Whatman No. 1 filter, and the filtrates were then sequentially extracted three times with an equal volume of hexane followed by ethyl acetate using a separating funnel. Using a rotary evaporator (IKA^®^ RV 10, Germany), the extracted fractions were evaporated below 401°C to obtain concentrates of hexane and ethyl acetate. The crude extracts of FOP were then added to 100 ml PDB and inoculated with 2–3 agar disks of *A. niger* aseptically (Figure 1). Control was maintained with mycelial disks of *A. niger* and FOP crude extracts separately. Flasks were incubated for 15 days at 26 ± 1°C. Following liquid–liquid partitioning, cultures were re-filtered and then extracted three more times using hexane and ethyl acetate. Anhydrous sodium sulfate (10 g) was passed through several solvent layers to get rid of any remaining moisture. Concentrated extract fractions were employed for GC–MS analysis.

#### 2.7.2. GC–MS analysis

The concentrated extract fractions were dissolved separately in GC–MS grade solvents, *viz.*, ethyl acetate (2 ml) and hexane (2 ml) and filtered with a 0.45 μm membrane (Millipore, Billerica, MA). Each sample was analyzed on a 559°C Agilent GC–MS (Agilent Technologies^®^, USA) to determine the volatile organic components. The mass spectrometer detected the components after they had been separated by an Agilent HP-5MS column (30 m, 0.25 mm, film thickness 0.25 m). As a carrier gas, helium gas (>99.99% purity) was used at a flow rate of 1 ml/minand a pressure of 10 psi. Each sample (1 L) was injected into the gas chromatograph (GC) using an in-built auto-injector with a 20:1 split ratio. A GC–MS temperature program was created that began at 40°C and enhanced at a rate of 3°C min^−1^ to reach 130°C, then held for 2 min. The temperature also rose at a pace of 5°C min^−1^ until it reached 210°C and was held for 2 min. The temperature was then raised to 350°C by adding 10°C min^−1^. The samples were run for a period of 64 min. The MS acquisition parameters were set to the following values: solvent delay of 2 min, E.M. voltage of 1,214 V, ion source temperature of 200°C, electron ionization of 70 eV, transfer line temperature of 200°C, and full scan mode of 50–550 AMU. Volatile organic components were characterized by matching them with NIST (National Institute of Standards and Technology, USA) mass spectral library data, corresponding retention index (RI), and mass fragmentation pattern (Kumar et al., 2021b).

### 2.8. Statistical analysis

To assess differences in parameter values, the data were analyzed using ANOVA in SPSS version 20.0 statistical software (SPSS, SAS Institute, USA). The experiments were repeated two times, with three replications of each treatment. Duncan's multiple range test was used to determine differences between treatments at a 5% level of significance. VENNY 2.1.0 (http://bioinfogp.cnb.csic.es/tools/venny/index.html) and SRplot (Science and Research Online Plot) (https://bioinformatics.com.cn) were used to statistically analyze the GC–MS data and for creating Venn diagrams and heat maps (Esmail et al., 2022; Zhou et al., 2022).

## 3. Results

### 3.1. Morphological identification of *A. niger* strains

All 11 strains expressed rapid growth on the PDA medium, mycelial growth was uniform, and colonies were initially white but later turned dark brown to black after incubation for 7 days (Supplementary Figure 1). The AN-4 strain initially formed a cream color colony, but after a few days, it turned black. The walls of the conidiophores were smooth, hyaline, and sometimes dark toward the vesicle. A biseriate conidial head with phialides was observed, the head of the conidia was brownish-black, and the conidia were globose or sub-globose, brown to black, rough, and irregular (Figure 1). The size of the conidia was measured, which varied from 3.4 μm (AN-3) to 4.3 μm (AN-8). All these growth and microscopic characteristics signify that the strains belong to the *A. niger* group (McClenny, 2005; Diba et al., 2007) and are listed in Table 1.

### 3.2. Molecular identification and phylogenetic analysis

**Figure 2:**
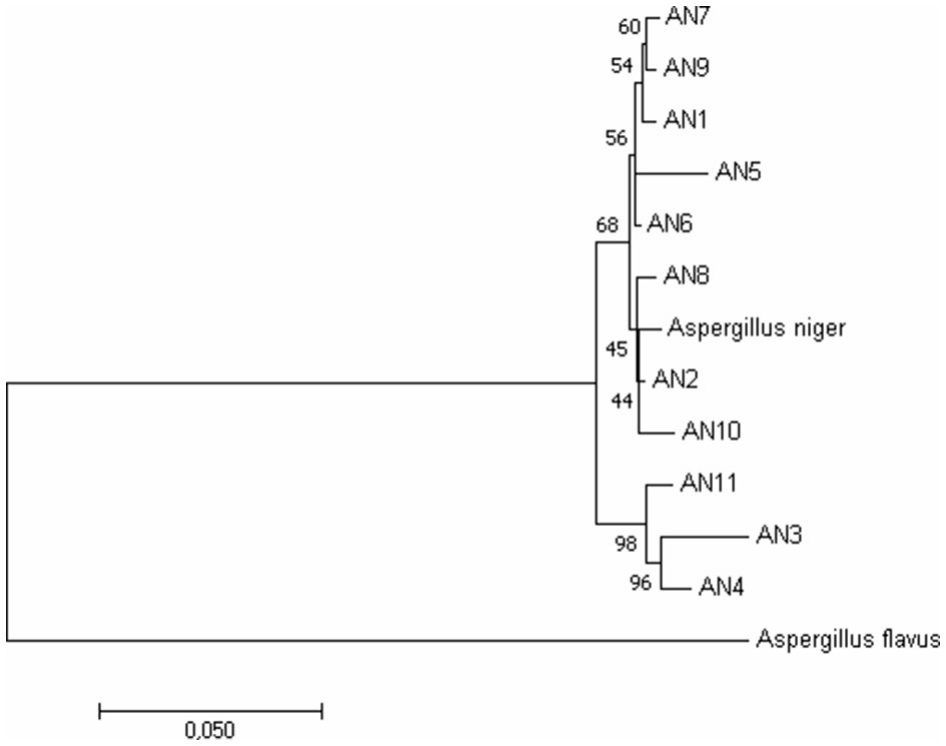
Phylogenetic relationship among *A. niger* isolates based on ITS, β-tubulin, and calmodulin genes by the Maximum Likelihood method. Bootstrap analysis was done with 1,000 replicates and values at nodes of branches refer to the number of times (%) the branching is supported.

To further confirm morphological identification, the DNA was isolated, and a PCR-based molecular study was carried out using the ITS, β-tubulin, and calmodulin genes. The amplified products were separated on agarose gel through gel electrophoresis, purified, sequenced, and analyzed for their quality through Bio-Edit software. The sequences were further submitted to GenBank, and accession numbers were obtained (Table 1). These 11 *A. niger* strains' taxonomic identity was established by NCBI blast and the alignments and phylogenetic analyses (Figure 2).

### 3.3. *In vitro* antagonistic assays

**Figure 3:**
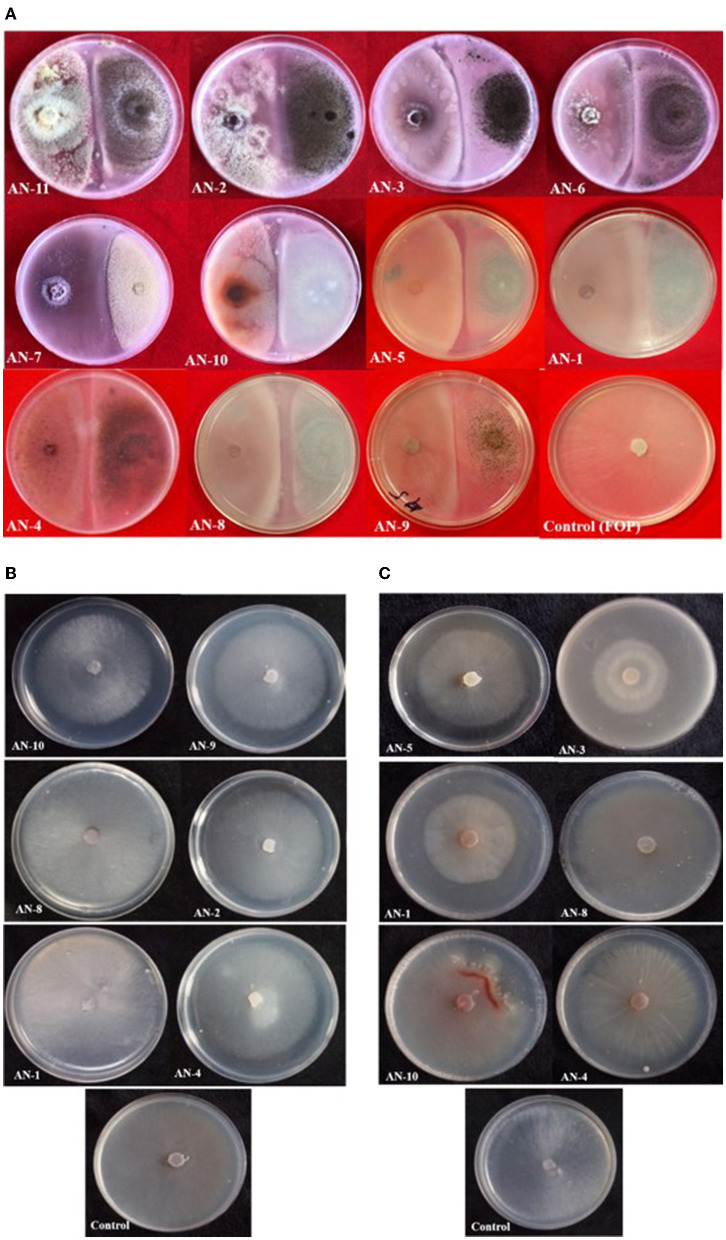
Growth inhibition of *F. oxysporum* f. sp. *psidii* by *A. niger* strains under *in vitro* conditions after 7 days of incubation on potato dextrose agar (PDA) media. **(A)** Dual culture method. **(B)** Volatile assay. **(C)** Non-volatile assay.

Under a dual culture assay, the mycelial growth of FOP was significantly inhibited by 11 *A. niger* strains, with percent inhibition ranging from 64.7% to 100% (*F* = 99.62, *P* < 0.05). Among them, four *A. niger* strains, *viz*., AN-2, AN-3, AN-6, and AN-11, showed 100% mycelial growth inhibition (Table 2, Figure 3A) after 7 days post-inoculation. The volatile compounds produced by *A. niger* strains showed 11.61–26.19% of mycelial inhibition (*F* = 10.19, *P* < 0.05) (Table 2, Figure 3B). AN-11 showed the maximum (26.19%) growth inhibition of the test pathogen, followed by AN-2 (22.62%) and AN-6 (22.61%). Under the non-volatile method, maximum inhibition was recorded in AN-11 (75.29%), and five strains (AN-1, AN-2, AN-6, AN-7, and AN-9) showed moderate growth inhibition ranging from 50 to 75%. The remaining five *A. niger* strains (AN-3, AN-4, AN-5, AN-8, and AN-10) showed low antagonism levels of 4.7–49.41% inhibition (*F* = 207.71, *P* < 0.05) (Table 2, Figure 3C) after 7 days of incubation. The collective effect of the *in vitro* bio-control assay of *A. niger* strains against FOP exhibited AN-11 as the most potential one (67.16%), followed by AN-6 (64.01%) and AN-2 (60.48%). Effective inhibition of the test pathogen was found in the dual culture method followed by the non-volatile method but very low percent mycelial inhibition was observed in the volatile method. The *A. niger* strains were divided into three groups based on their *in vitro* potentiality against *F. oxysporum* f. sp. *psidii* (Table 3).

**Table 2:** Inhibition of mycelial growth of *F. oxysporum* f. sp. *psidii* by *A. niger* strains under *in vitro* conditions.

**Isolate**	Dual culture^*^	Volatile^*^	Non-volatile^*^	
AN-1	90.58^b^	14.28^b^	67.05^b^	57.30
AN-2	100^a^	22.62^a^	58.82^d^	60.48
AN-3	100^a^	20.24^a^	49.41^f^	56.55
AN-4	76.47^c^	16.66^b^	20.00^h^	37.71
AN-5	83.52^c^	19.04^b^	38.82^g^	47.13
AN-6	100^a^	22.61^a^	69.41^a^	64.01
AN-7	78.82^c^	16.66^b^	62.35^c^	52.61
AN-8	64.70^d^	11.61^c^	4.70^j^	27.00
AN-9	70.00^d^	23.80^a^	52.9^e^	48.90
AN-10	89.41^b^	21.42^a^	5.80^i^	38.88
AN-11	100^a^	26.19^a^	75.29^a^	67.16
CD @ 5%	8.012	6.043	5.036	
SE	3.88	2.92	2.43	

* Different letters after values are significantly different at p ≤ 0.05.

**Table 3:** Grouping of *A. niger* strains based on percent inhibition of *F. oxysporum* f. sp. *psidii* under *in vitro* conditions.

**Inhibition**	**Group**	**% Inhibition of** ***F. oxysporum*** **f. sp**. ***psidii***		
		**Dual culture method**	**Volatile assay**	**Non-volatile assay**
High	Group 1 (75–100% inhibition)	AN1, AN2, AN3, AN4, AN5, AN6, AN7, AN9, AN10 and AN11	–	AN11
	Number of isolates	10	0	1
Moderate	Group 2 (50–75% inhibition)	AN8	–	AN1, AN2, AN6, AN7, and AN9
	Number of isolates	1	0	5
Low	Group 3 (<50 % inhibition)	–	AN1, AN2, AN3, AN4, AN5, AN6, AN7, AN8, AN9, AN10 and AN11	AN3, AN4, AN5, AN8 and AN10
	Number of isolates	0	11	5

### 3.4. *In planta* bio-control assay

**Figure 4:**
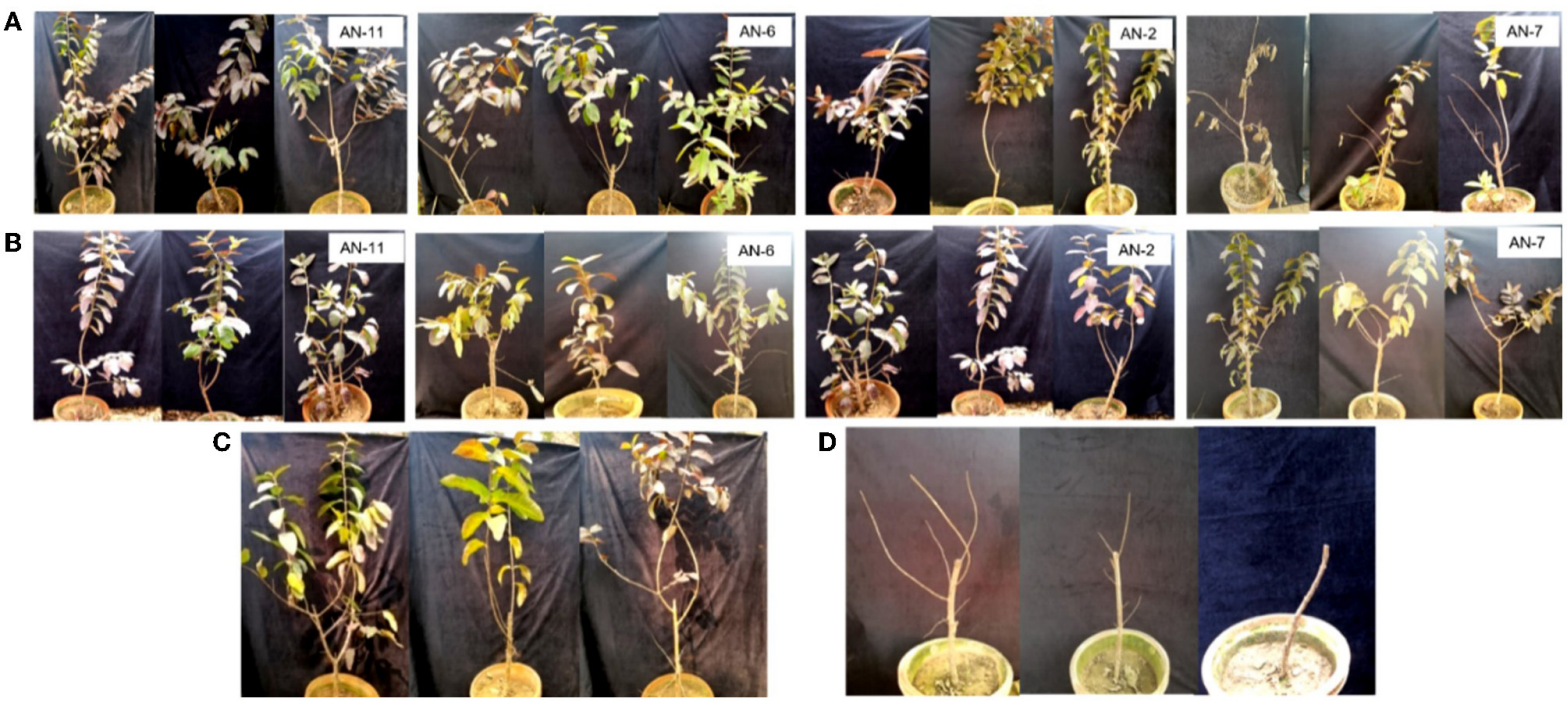
Effect of *A. niger* strains on guava wilt under greenhouse conditions (three replications per treatment). **(A)** Post-inoculation application (AN-11, AN-6, AN-2, and AN-7). **(B)** Pre-inoculation application (AN-11, AN-6, AN-2, and AN-7). **(C)** Wilted plants treated with Carbendazim @ 0.2%. **(D)** Guava plants inoculate with *F. oxysporum* f. sp. *psidii* only.

**Figure 5:**
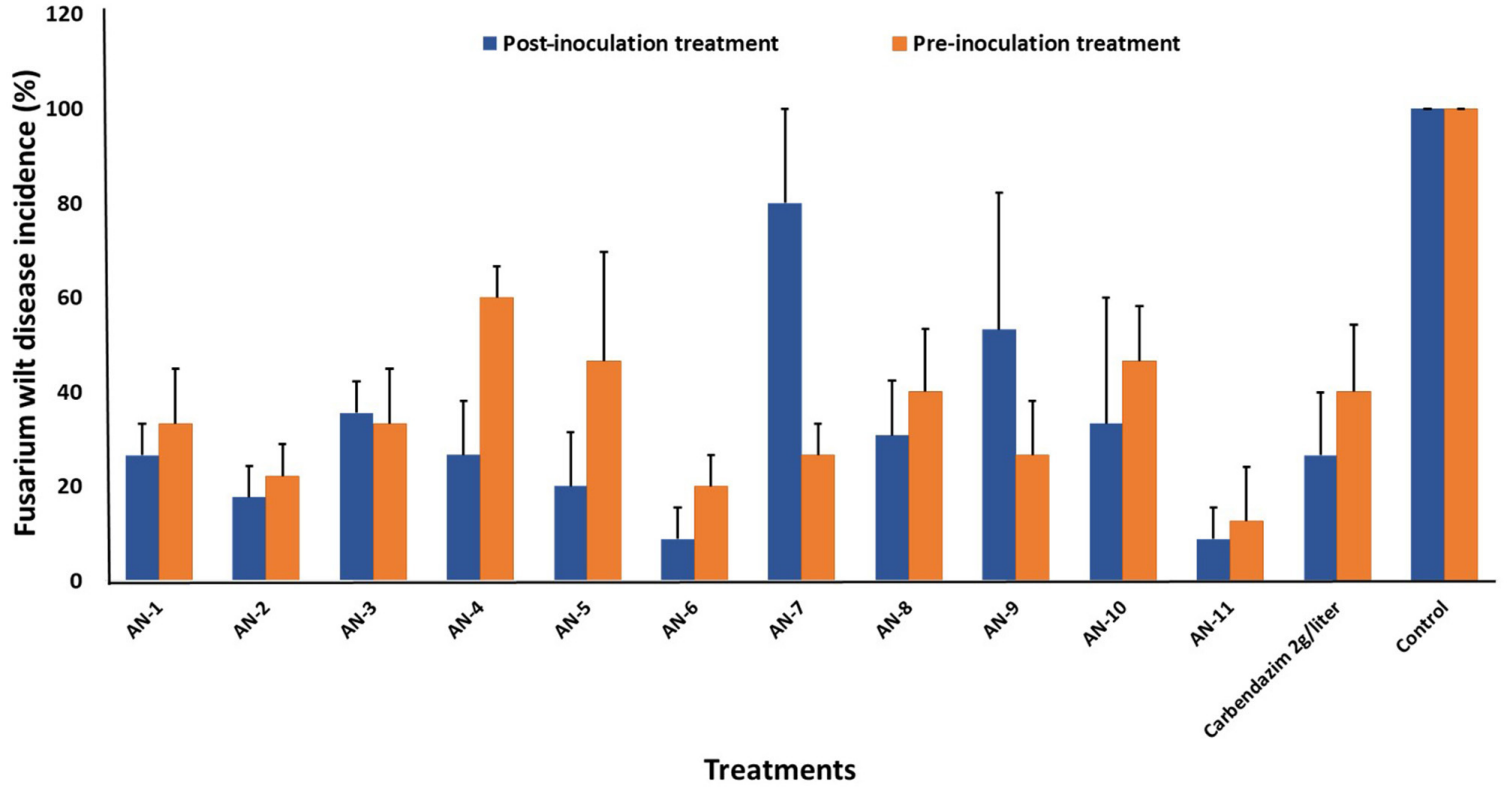
Effect of *A. niger* strains on percent wilt incidence of guava seedlings during post and pre-inoculation treatment of *F. oxysporum* f. sp. *psidii* (FOP) under greenhouse conditions. Bars represent the mean values obtained in three independent repeats and standard error is indicated by error bars. AN represents *A. niger*.

*A. niger* strains, *viz*., AN-11 (12.6%), AN-6 (20%), AN-2 (22.2%), and AN-7 (26.64%), showed lower wilting incidence when compared with control when applied as pre-inoculation treatment (*F* = 4.44, df = 24, *P* < 0.05) (Figures 4B, 5). Positive control plants inoculated with only FOP exhibited complete wilting and could not recover (Figure 4D). The AN-11 strain was found to be the best treatment, showing an 87.4% reduction of wilt, followed by the AN-6 strain (80%), whereas treatment with Carbendazim (2 g/l) showed a 60% reduction in disease over control (Figure 4C). The efficiency of *A. niger* strains was assessed by post-inoculation treatment with FOP. The lowest percent of wilting was observed in the treatment with AN-6 (8.78%) and AN-11 (8.78%), followed by AN-2 (17.7%) and AN-5 (20%) (*F* = 4.41, df = 24, *P* < 0.05) (Figures 4A, 5). Treatment with strain AN-7 showed the highest disease incidence (80%) (Figure 4A). Notably, AN-2, AN-5, AN-6, and AN-11 strains were found to be significantly effective, causing an 80–91% reduction in wilt incidence and maximum recovery from the disease under field conditions. The control treatment with Carbendazim resulted in a 73% disease reduction over the positive control (Figure 4C). Furthermore, the most potent strain AN-11 was used to study the antagonistic nature and to characterize the secondary metabolites during interaction with *F. oxysporum* f. sp. *psidii* (FOP).

### 3.5. Hyphal interactions between antagonist and pathogen

**Figure 6:**
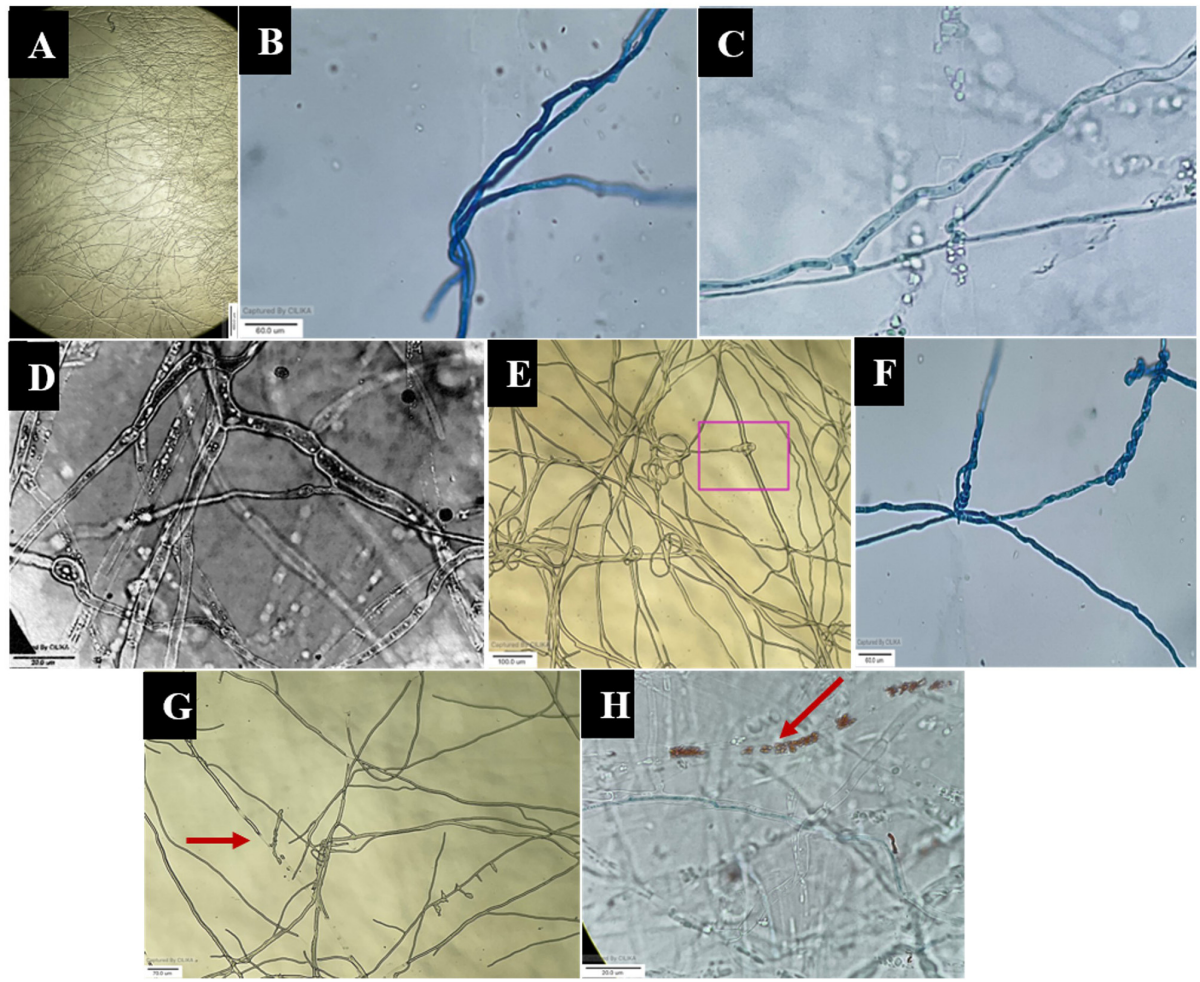
Microscopic view of mycoparasitic nature of *A. niger* (AN) on *F. oxysporum* f. sp. *psidii* (FOP). **(A)** Interaction zone; **(B)** Parallel growing of hyphae of *A. niger* (AN) to *F. oxysporum* f. sp. *psidii* (FOP) hyphae; **(C, D)** Initial contact and penetration of hyphae forming appressoria-like structure; **(E, F)** Hook or pincer-like structure (coiling) formed around *F. oxysporum* f. sp. *psidii* by *A. niger*; **(G, H)** Red arrow indicates plasmolysis leading to the death of hyphae (*F. oxysporum* f. sp. *psidii*).

**Figure 7:**
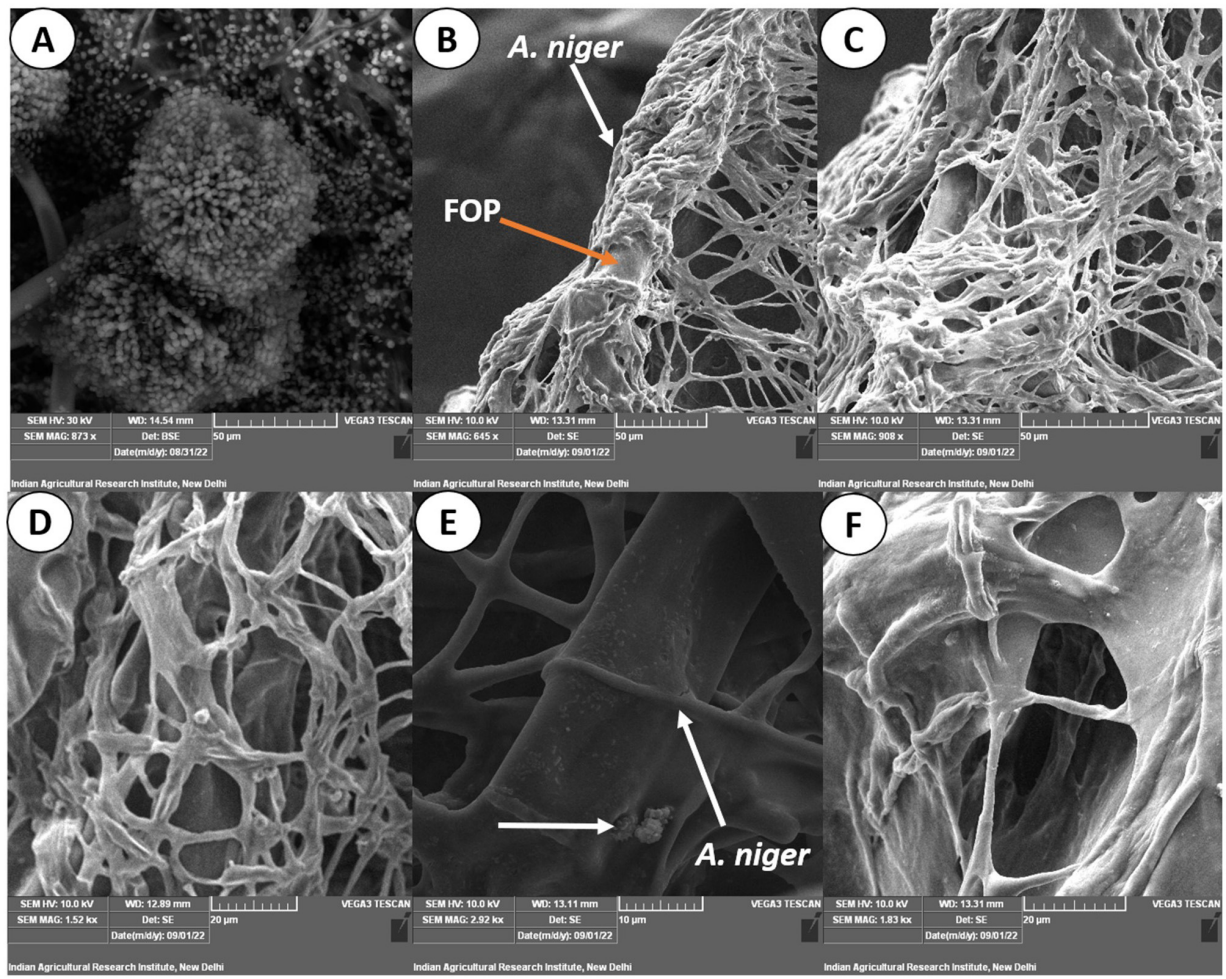
Scanning electron micrograph on mycoparasitism of *F. oxysporum* f. sp. *psidii* by *A. niger*. **(A)** Fruiting body of the bio-control *A. niger*; **(B–D)** Hyphal network of *A. niger* (white arrow) surrounding the pathogen hyphae (orange arrow); **(E)** Arrow shows coiling (pincer-like structure) and dissolution of pathogen hyphae by *A. niger*; **(F)** Plasmolysis and death of pathogen.

The images obtained with the compound and scanning microscopes were analyzed to identify structural changes during the interaction (Figures 6, 7). The antagonistic growth was found to be faster, and the hyphae were characterized as smaller in diameter with respect to the pathogen *F. oxysporum* f. sp. *psidii*. Initially, the antagonist grows parallel to the pathogen hyphae and later, it forms initial contact through a peg-like structure that helps in attachment. Furthermore, the perforation (hole) was formed in the pathogen hyphae. Most importantly, a hook or pincer-like structure that coils around and restricts the growth of the pathogen was observed, resulting in the death of hyphae. These results illustrate the antagonistic nature of a bio-control *A. niger* through mycoparasitism.

### 3.6. Chemical analysis

To confirm the potential bio-control efficacy of the VOCs from the potential *A. niger* strain, chemical analysis was performed using GC–MS. From the hexane soluble fraction of the bio-control fungal strain, identified components were grouped into different chemical classes based on their functional groups, which showed the highest content of hydrocarbons (22.88%), followed by acids (5.23%), esters (0.87%), and alcohol (0.81%) in *A. niger*. Similarly, in the interaction of *A. niger* with the hexane fraction of FOP, hydrocarbons (14.04%) secured the highest position, followed by esters (10.43%) and acids (8.96%). However, a total of 23 compounds were identified in *Fusarium* FOP, including hexadecanoic acid methyl ester (8.54%), 9-octadecanoic acid methyl ester (7.38%), dodecane (3.50%), and tridecane (3.41%). However, 23 VOCs, including octadecene (3.98%), hexadecane (3.95%), tetradecane (3.54%), and 1.3-dimethylcyclopentane (3.50%), were found in the bio-control fungi, *A. niger*. Interestingly, from the interacted plates, 30 VOCs were detected and characterized as hexadecanoic acid methyl ester (4.41%), 10-octadecanoic acid methyl ester (3.79%), dodecane (3.21%), and undecane (3.19%). In addition, minor compounds were also detected such as gibepyrone A (0.15%), 3-methylundecane (0.36%), and citroflex A (0.38%) from the interacted plate. The list of VOCs identified in the hexane fraction is listed in Table 4.

**Table 4:** List of volatile organic compounds (VOCs) in the hexane fraction during *F. oxysporum* f. sp. *psidii* and *A. niger* interaction, determined by GC–MS.

**Sl. No**	**Compound**	**Organic Group**	**Chemical formula**	***F. oxysporum*** **f. sp**. ***psidii*** **(control)**		***A. niger*** **(control)**		***F. oxysporum*** **f. sp**. ***psidii*** **and** ***A. niger*** **interaction**	
				**RT** ^*^	**Area %**	**RT** ^*^	**Area %**	**RT** ^*^	**Area %**
1	1,3-dimethylcyclopentane	Hydrocarbon	C_7_H_14_	3.71	1.31 ± 0.28	3.71	3.5 ± 1.39	–	–
2	Methyl benzol	Alcohol	C_8_H_8_O_2_	5.05	0.22 ± 0.08	–	–	–	–
3	Undecane	Hydrocarbon	C_11_H_24_	21.68	1.39 ± 0.62	21.572	0.28 ± 0.12	21.91	3.19 ± 0.47
4	Tetramethyl benzene	Hydrocarbon	C_10_H_14_	24.85	0.26 ± 0.11	–	–	22.61	1.62 ± 0.32
5	Dodecane	Hydrocarbon	C_12_H_26_	28.61	3.5 ± 2.01	28.429	1.15 ± 0.71	28.741	3.21 ± 0.38
6	2,6-dimethyl undecane	Hydrocarbon	C_13_H_28_	29.39	0.74 ± 0.38	–	–	29.47	0.66 ± 0.12
7	2-methyl-dodecane	Hydrocarbon	C_13_H_28_	32.67	0.24 ± 0.09	–	–	32.71	0.18 ± 0.06
8	Tridecane	Hydrocarbon	C_13_H_28_	35.27	3.41 ± 1.15	35.055	0.4 ± 0.19	–	–
9	Tetradecane	Hydrocarbon	C_14_H_30_	41.5	0.62 ± 0.16	41.619	3.54 ± 1.46	41.538	0.47 ± 0.11
10	Tetradecanoic acid methyl ester	Ester	C_15_H_30_O_2_	59.03	0.28 ± 0.73	–	–	59.032	0.16 ± 0.04
11	Hexadecanoic acid methyl ester	Ester	C_17_H_34_O_2_	62.94	8.54 ± 1.64	–	–	62.929	4.41 ± 1.94
12	*cis*-9-hexadecenoic acid	Acid	C_16_H_30_O_2_	63.23	0.32 ± 0.02	63.20	0.54 ± 0.22	63.282	0.49 ± 0.13
13	Hexadecanoic acid	Acid	C_16_H_32_O_2_	63.58	1.51 ± 0.87	63.52	1.65 ± 1.04	63.648	1.86 ± 0.89
14	2-tetradecene	Hydrocarbon	C_14_H_28_	64.54	0.27 ± 0.06	40.95	0.26 ± 0.05	–	–
15	Cyclotetracosane	Hydrocarbon	C_24_H_48_	64.71	1.43 ± 0.11	–	–	–	–
16	Methyl-octadecadienoate	Ester	C_19_H_34_O_2_	64.93	2.59 ± 0.31	–	–	–	–
17	9-octadecenoic acid methyl ester	Ester	C_19_H_36_O_2_	65.06	7.38 ± 2.05	–	–	–	–
18	Octadecanoic acid methyl ester	Ester	C_19_H_38_O_2_	65.25	1.35 ± 0.24	65.23	0.13 ± 0.04	65.257	0.65 ± 0.03
19	6-Octadecenoic acid	Acid	C_18_H_34_O_2_	65.51	2.59 ± 0.49	65.48	1.14 ± 0.82	65.597	2.06 ± 1.38
20	9-Tricosene	Hydrocarbon	C_23_H_46_	65.65	1.29 ± 0.08	61.048	0.18 ± 0.01	–	–
21	Octadecanoic acid	Acid	C_18_H_36_O_2_	65.71	0.54 ± 0.07	–	–	65.75	0.53 ± 0.07
22	1,2-dimethylcyclopentane	Hydrocarbon	C_7_H_14_	–	–	3.77	1.84 ± 0.38	–	–
23	Hexadecane	Hydrocarbon	C_16_H_34_	–	–	47.24	3.95 ± 1.93	–	–
24	2,4-dibutyl phenol	Phenol	C_14_H_22_O	–	–	53.146	0.54 ± 0.08	–	–
25	3-methylpentadecane	Hydrocarbon	C_16_H_34_	–	–	51.639	0.3 ± 0.17	–	–
26	Heptadecane	Hydrocarbon	C_17_H_36_	–	–	58.238	0.21 ± 0.02	–	–
27	4-formyl-2,6-di-tert-butylphenol	Phenol	C_14_H_22_O	–	–	59.983	0.38 ± 0.14	–	–
28	15-methyl-heptadecane	Hydrocarbon	C_18_H_38_	–	–	60.118	0.39 ± 0.18	–	–
29	Tetradecanoic acid	Acid	C_14_H_28_O_2_	–	–	60.24	0.25 ± 0.04	60.274	0.1 ± 0.05
30	Octadecane	Hydrocarbon	C_18_H_38_	–	–	60.78	3.98 ± 2.07	60.72	0.19 ± 0.13
31	Eicosane	Hydrocarbon	C_20_H_42_	–	–	63.805	2.9 ± 0.83	63.77	0.19 ± 0.06
32	1-heneicosanol	Alcohol	C_21_H_44_O	–	–	64.64	0.47 ± 0.12	–	–
33	Butyl-2-ethyloctahydro epoxy indenol	Alcohol	C_15_H_26_O_2_	–	–	64.897	0.34 ± 0.09	–	–
34	10-octadecenoic acid methyl ester	Ester	C_19_H_36_O_2_	–	–	64.97	0.74 ± 0.32	65.06	3.79 ± 2.01
35	Nonane	Hydrocarbon	C9H_2_0	–	–	–	–	9.71	0.25 ± 0.18
36	Dimethyl octane	Hydrocarbon	C_10_H_22_	–	–	–	–	11.36	0.2 ± 0.04
37	3-ethyl-2-methyl-heptane	Hydrocarbon	C_10_H_22_	–	–	–	–	11.71	0.12 ± 0.07
38	Trimethyl-benzene	Aromatic Hydrocarbon	C_9_H_12_	–	–	–	–	13.33	1.31 ± 0.74
39	Decane	Hydrocarbon	C10H__2_2_	–	–	–	–	15.34	1.49 ± 0.13
40	4-methyldecane	Hydrocarbon	C_11_H_24_	–	–	–	–	19.033	0.27 ± 0.09
41	2-ethylnonane	Hydrocarbon	C_11_H_24_O_3_	–	–	–	–	19.69	0.33 ± 0.02
42	3-methylundecane	Hydrocarbon	C_12_H_26_	–	–	–	–	26.508	0.36 ± 0.04
43	Gibepyrone A	Ketone	C_10_H_12_O_2_	–	–	–	–	48.136	0.15 ± 0.01
44	Citroflex A	Carbonyl group	C_20_H_34_O_8_	–	–	–	–	60.634	0.38 ± 0.14
45	Octadecadienoic acid methyl ester	Ester	C_19_H_34_O_2_	–	–	–	–	64.938	1.42 ± 0.81
46	9-octadecenoic acid	Acid	C_18_H_34_O_2_	–	–	–	–	65.597	2.06 ± 0.96
**Chemical groups**				**Content (%)**					
Hydrocarbons				14.46		22.88		14.04	
Esters				20.14		0.87		10.43	
Alcohol				0.22		0.81		–	
Acids				4.96		5.23		8.96	
Phenols				–		0.92		–	
Ketone				–		–		0.15	
Carbonyl group				–		–		0.38	
Total				39.78		29.90		33.96	

* RT, retention time (min) of each volatile compound eluted through the HP 5MS column in GC–MS.

**Figure 8:**
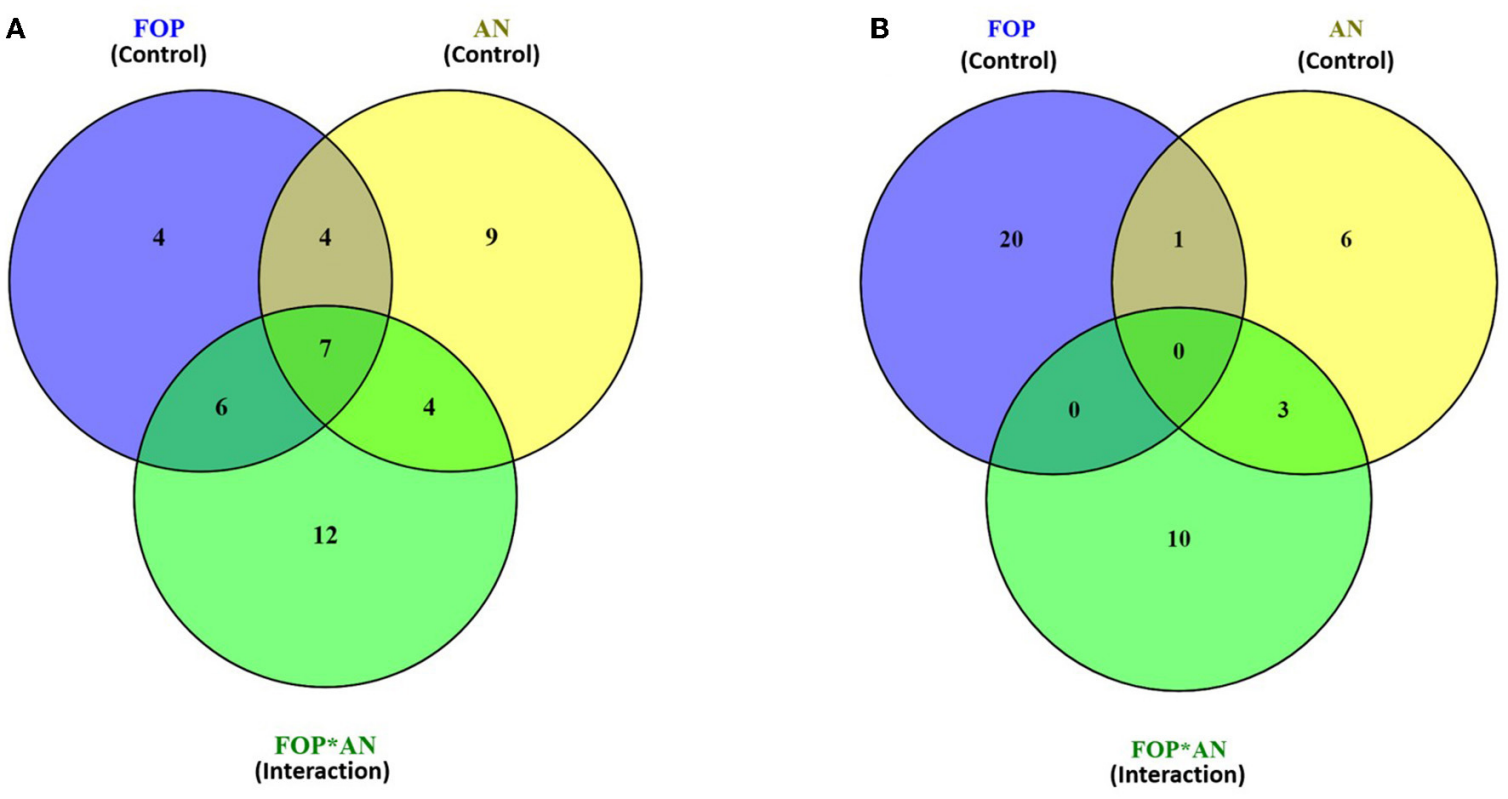
Venn diagram representing the compounds identified through GC–MS analysis of **(A)** hexane and **(B)** ethyl acetate fractions during *F. oxysporum* f. sp. *psidii* and *A. niger* interaction.

**Figure 9:**
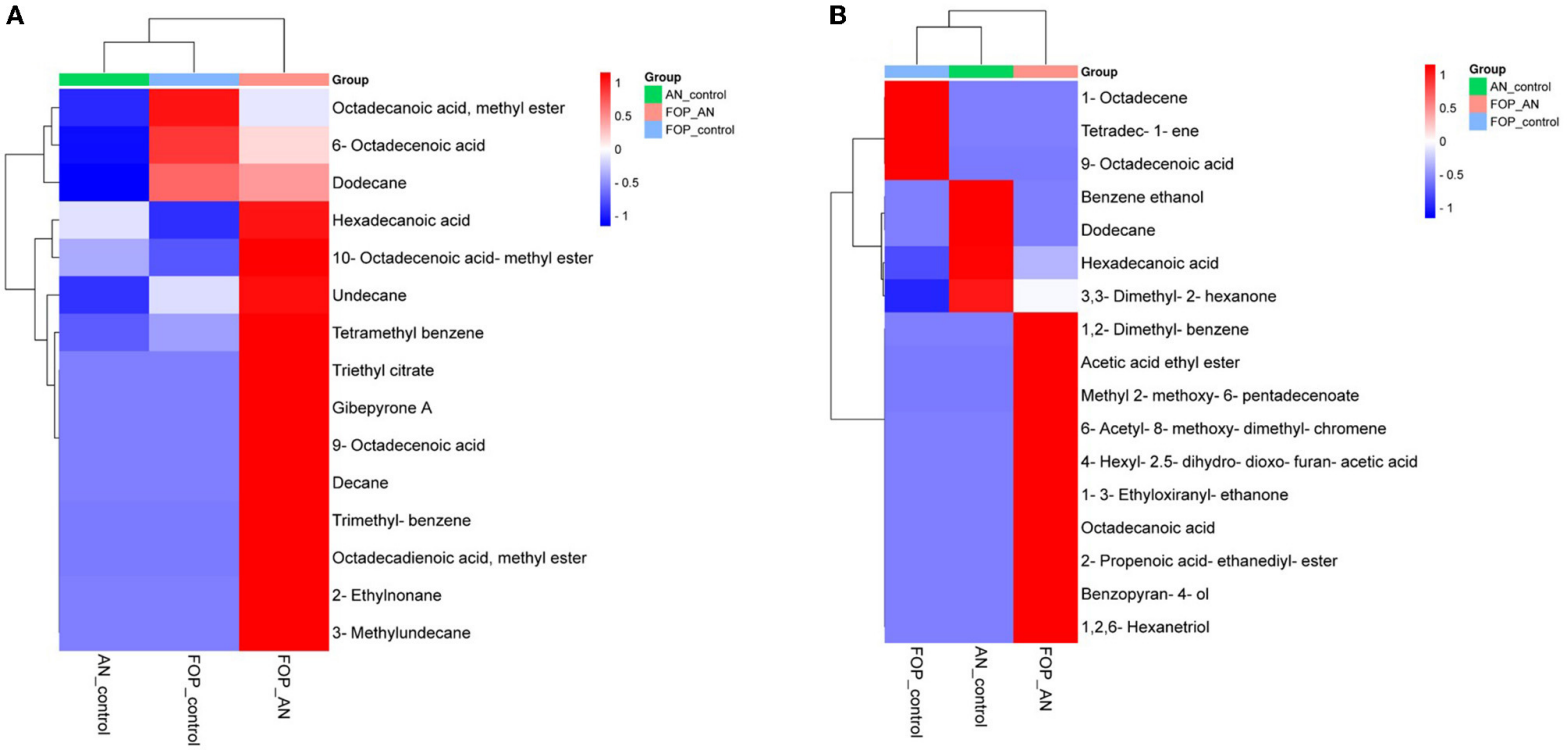
Heat map clustering of volatile organic compounds (VOCs) profiles from *A. niger* (AN) and *F. oxysporum* f. sp. *psidii* (FOP) and their interactions in **(A)** hexane and **(B)** ethyl acetate fractions.

In the ethyl acetate soluble fraction of *Fusarium* FOP, predominant volatile compounds were identified and grouped into their respective chemical classes, which showed the highest content of hydrocarbons (35.52%), followed by acids (13.18%), esters (5.25%), and phenols (3.40%). Among these, *n*-hexadecanoic acid (4.37%) and 3-methoxy-2,4,5-trimethylphenol (3.40%) were most abundant in FOP. Surprisingly, alcohol (12.91%), acids (7.6%), and hydrocarbons (1.44%) were detected in the bio-control fungal strain, *A. niger*, where dodecene (13.92%), benzene ethanol (10.74%), hexadecanoic acid (7.15%), 2-2-furylethyl-N-methylaniline (3.62%), and 3-diethynyl-9,10-dimethoxyanthracene (2.29%) represented the highest components of the ethyl acetate fraction. Consequently, interaction treatment of *A. niger* and FOP exhibited an abundance of esters (24.08%) and alcohol (21.33%) including certain acids (3.05%). Here, acetic acid ethyl ester (17.32%), benzopyron-4-ol (12.17%), 1,2,6-hexanetriol (7.16%), and 2-propenoic acid ethanediyl ester (2.95%) were found as the major VOCs. In addition, minor compounds, namely, octadecanoic acid (1.11%), 1-(3-ethyloxiranyl) ethanone (0.98%), 6-acetyl-8-methoxy dimethyl chromene (0.96%), and 4-hexyl-2,5-dihydro dioxo furan acetic acid (0.19%) were also detected in the interaction treatment. The list of VOCs identified in the ethyl acetate fraction is listed in Table 5. The total ion chromatograms of the hexane and ethyl acetate fractions are shown in Supplementary Figures 2–7. Furthermore, Venn diagrams and heat maps were generated in the current experiment to compare the visualization and interpretation of changes in the VOCs profiling during interaction (Figures 8, 9).

**Table 5:** List of volatile organic compounds (VOCs) in the ethyl acetate fraction during *F. oxysporum* f. sp. *psidii* and *A. niger* interaction, determined by GC–MS.

**Sl. No**	**Compound**	**Organic Group**	**Chemical formula**	***F. oxysporum*** **f. sp**. ***psidii*** **(control)**		***A. niger*** **(control)**		***F. oxysporum*** **f. sp**. ***psidii*** **and** ***A. niger*** **interaction**	
				**RT** ^*^	**Area %**	**RT** ^*^	**Area %**	**RT** ^*^	**Area %**
1	Ethyl propanoate	Ester	C_5_H_10_O_2_	5.13	0.18 ± 0.12	–	–	–	–
2	3-hydroxy-ethyl butyrate	Ester	C_6_H_12_O	12.25	1.3 ± 0.19	–	–	–	–
3	2-dodecene	Hydrocarbon	C_12_H_24_	27.90	0.98 ± 0.22	–	–	–	–
4	*n*-dodecane	Hydrocarbon	C_12_H_26_	28.39	0.15 ± 0.02	17.21	13.92 ± 3.36	–	–
5	*n*-tridecane	Hydrocarbon	C_13_H_28_	35.04	0.02 ± 0.01	–	–	–	–
6	Tetradec-1-ene	Hydrocarbon	C_14_H_28_	41.36	7.89 ± 0.95	–	–	–	–
7	*n*-tetradecane	Hydrocarbon	C_14_H_30_	41.62	0.54 ± 0.18	–	–	–	–
8	α-hexadecene	Hydrocarbon	C_16_H_32_	53.38	2.81 ± 0.16	–	–	–	–
9	*n*-hexadecane	Hydrocarbon	C16H34	53.60	0.62 ± 0.03	–	–	–	–
10	3-methoxy-2,4,5-trimethylphenol	Phenol	C_10_H_14_O_2_	54.32	3.4 ± 0.56	–	–	–	–
11	Dodecyl acrylate	Ester	C_15_H_28_O_2_	58.14	0.97 ± 0.04	–	–	–	–
12	Myristate	Ester	C_15_H_30_O_2_	60.53	0.97 ± 0.11	–	–	–	–
13	1-octadecene	Hydrocarbon	C_18_H_36_	60.78	22.26 ± 2.2	–	–	–	–
14	2-tetradecene	Hydrocarbon	C_14_H_28_	60.83	0.07 ± 0.02	–	–	–	–
15	Cyclopentadecane	Hydrocarbon	C_15_H_30_	62.60	0.21 ± 0.07	–	–	–	–
16	Hexadecanoate	Ester	C_17_H_34_O_2_	62.84	1.24 ± 0.92	–	–	–	–
17	3-eicosene	Hydrocarbon	C_20_H_4_O	63.15	0.06 ± 0.05	–	–	–	–
18	*n*-hexadecanoic acid	Acid	C_16_H_32_O_2_	64.00	4.37 ± 1.84	–	–	–	–
19	1-eicosene	Hydrocarbon	C_20_H_4_O	64.61	0.1 ± 0.06	–	–	–	–
20	9-octadecenoic acid	Acid	C_18_H_34_O_2_	64.85	8.81 ± 2.17	–	–	–	–
21	10-octadecenoic acid methyl ester	Ester	C_19_H_36_O_2_	64.99	0.59 ± 0.03	–	–	–	–
22	Pentamethyl-2,3-dihydro indene	Hydrocarbon	C_14_H_2_O	–	–	17.82	3.3 ± 0.24	–	–
23	Tetradecane	Hydrocarbon	C_14_H_3_O	–	–	28.65	1.58 ± 0.87	–	–
24	Benzene ethanol	Alcohol	C_8_H_10_O	–	–	33.59	10.74 ± 1.48	–	–
25	Hexadecane	Hydrocarbon	C_16_H_34_	–	–	38.60	0.94 ± 0.31	–	–
26	2-2-furylethyl-*N*-methylaniline	Nitrogenous compound	C_7_H_8_FN	–	–	43.78	3.62 ± 1.46	–	–
27	4-ethylbenzoic acid	Acid	C_9_H_10_O_2_	–	–	52.32	0.45 ± 0.09	–	–
28	Acetic acid ethyl ester	Ester	C_4_H_8_O_2_	–	–	–	–	7.28	17.32 ± 2.19
29	3,3-dimethyl-2-hexanone	Alcohol	C_8_H_16_O	–	–	10.01	2.17 ± 0.94	9.62	1.02 ± 0.24
30	1,2-dimethyl benzene	Hydrocarbon	C_8_H_10_	–	–	–	–	10.1	0.56 ± 0.02
31	Methyl-2-methoxy-6-pentadecenoate	Ester	C_10_H_12_O_3_	–	–	–	–	13.18	3.81 ± 1.22
32	1-(3-ethyloxiranyl) ethanone	Alcohol	C_6_H_10_O_2_	–	–	–	–	24.75	0.98 ± 0.07
33	Benzopyran-4-ol	Alcohol	C_9_H_8_O_2_	–	–	–	–	39.14	12.17 ± 3.16
34	6-acetyl-8-methoxy dimethyl chromene	Hydrocarbon	C_14_H_16_O_3_	–	–	–	–	39.86	0.9 ± 0.18
35	1,2,6-hexanetriol	Alcohol	C_6_H_14_O_3_	–	–	–	–	40.5	7.16 ± 1.92
36	Hexadecanoic acid	Acid	C_16_H_32_O_2_	–	–	40.99	7.15 ± 1.45	41.96	1.75 ± 0.06
37	4-hexyl-2,5-dihydro dioxo furan acetic acid	Acid	C_12_H_16_O_5_	–	–	–	–	42.03	0.19 ± 0.11
38	2-propenoic acid ethanediyl ester	Ester	C_10_H_14_O_4_	–	–	–	–	42.11	2.95 ± 0.02
39	Octadecanoic acid	Acid	C_18_H_36_O_2_	–	–	–	–	48.29	1.11 ± 0.04
40	3-diethynyl-9,10-dimethoxyanthracene	Hydrocarbon	C_16_H_14_O_2_	–	–	55.39	2.29 ± 0.74	55.12	0.24 ±0.011
**Chemical groups**				**Content (%)**					
Hydrocarbons				35.52		1.44		1.76	
Esters				5.25		–		24.08	
Alcohol				–		12.91		21.33	
Acids				13.18		7.6		3.05	
Phenols				3.4		–		–	
Total				57.35		21.95		50.22	

* RT, retention time (min) of each volatile compound eluted through HP 5MS column in GC–MS.

## 4. Discussion

The biotic stresses are especially caused by fungal pathogens, resulting in the huge economic loss of agricultural and horticultural crops throughout the world. The production of guava is diminishing year by year due to wilt infections caused by *Fusarium* spp. Management is not successful in the field because of the soil-borne nature of pathogens. In recent days, the use of potential BCAs for the management of pests and diseases is because of their cost-effectiveness, sustainability, and eco-friendliness. The fungus *A. niger* is proven to be a potential BCA against many plant pathogens, including guava wilt disease. Considering this, our present study is undertaken to evaluate *A. niger* strains against the guava wilt pathogen.

### 4.1. Morphological and molecular characterization of *A. niger* strains

The study of *Aspergillus* species has always been a confusing and challenging task due to the complex and subtle differences between them (Silva et al., 2011). In our study, 11 strains used as antagonists were confirmed as *A. niger* based on morphological and molecular characteristics. The studied morphological characteristics, such as colony morphology, conidia, vesicle, and conidiophore nature, confirmed the previous reports (McClenny, 2005; Diba et al., 2007; Varga et al., 2011; Lanka et al., 2017; Atallah et al., 2022). The conventional morphological approaches may not perform well for the accurate identification of the genus *Aspergillus* up to the species level. The difficulties are mainly related to the identification of cryptic species, which are morphologically similar species. So, combining the morphological and molecular strategies will be an ideal tool for accurate identification. Due to its high level of sequence polymorphism, the ITS region has been considered, for a long time, the most efficient marker for the identification and detection of *Aspergillus* up to the species level (Nithiyaa et al., 2012; George and Ramteke, 2019; El-Shora et al., 2021). Housekeeping genes, particularly the β-tubulin and calmodulin genes, have proven to be highly beneficial in differentiating species belonging to section Nigri due to their species-specific traits (Samson et al., 2007; Nasri et al., 2015). The combined DNA sequence analysis of the ITS, β-tubulin, and calmodulin genes has shown a high degree of intraspecies discriminatory power for *A. niger* (Susca et al., 2013). Considering this, the combined DNA sequence and phylogenetic analysis carried out in our study confirmed that all the isolates belonged to *A. niger*. Overall, morphological and combined gene molecular analysis in the present study confirms 11 strains as *A. niger*.

### 4.2. Bio-efficacy test of *A. niger* strains against guava wilt pathogen

Furthermore, these 11 strains were tested against the most potent guava wilt pathogen, FOP, *A. niger*, as an effective bio-control agent. In the dual culture method, four strains exhibited 100% growth inhibition of FOP, and collectively, under *in vitro* assays, *A. niger* strain AN-11 (67.16%) was found to have the most potential, followed by AN-6 (64.01%) against the guava wilt pathogen. These results indicate that BCA *A. niger* inhibits the pathogen by various bio-control mechanisms, such as mycoparasitism, as well as by competing for space and nutrients. In addition, it produces non-volatile and volatile secondary metabolites, thereby restricting the growth of pathogens (Patibanda and Sen, 2005; Ferreira and Musumeci, 2021). *A. niger* has been found to be promising in inhibiting the guava wilt pathogen. The results are correlated with the previous studies, namely Dwivedi and Shukla (2002), Singh et al. (2003), Dwivedi and Dwivedi (2012), Naz et al. (2013), and Mandal et al. (2021). Sharma et al. (2011) tested *A. niger* against *Fusarium* wilt of tomato under *in vitro*, the highest mycelial inhibition of 74% was recorded, followed by *Trichoderma* spp., *Bacillus* spp. and, *Pencillium* spp. An *in planta* study carried out using guava pants (Allahabad Safeda) suggests that the application of *A. niger* to the soil near the root zone before the development of the disease will reduce the wilt incidence significantly, whereas, the control showed 100% wilt incidence with prominent symptoms. The wilted plants recovered from wilting within a few days after being treated with *A. niger. Aspergillus versicolor* exhibited a 99.2% reduction in *F. oxysporum* f. sp. *cumini* and also has the ability to survive in hot, arid soils (Israel and Lodha, 2005). An experiment conducted by Boughalleb-M'Hamdi et al. (2018) recorded the antifungal activity of *Aspergillus* spp. against *F. oxysporum* f. sp. *niveum* and *F. solani* f. sp. *cucurbitae*. Among six bioagents evaluated against *F. oxysporum* f. sp. *melangene*, maximum mycelial growth inhibition of 65.92% and pre-emergence seedling mortality of 11.33% were recorded upon application of *A. niger* (Govardhan et al., 2020). The *in vitro* and *in planta* bio-efficacy test against peach seedling decline was conducted by Mannai and Boughalleb-M'Hamdi (2022) and resulted in the mycelial growth inhibition of *F. oxysporum* (85.82%) by *Aspergillus candidus*. Similarly, *A. flavus* and *A. niger* were found more effective against *F. solani*, with more than 60% mycelial inhibition. Attia et al. (2022) evaluated four plant growth-promoting fungi (PGPF), namely *A. flavus, A. niger, Mucor circinelloides*, and *Pencillium oxalicum*, against the tomato wilt pathogen (*F. oxysporum*). The *A. niger* significantly reduced disease severity by 16.60% and gave a high level of protection of (86.35%), thus enhancing the growth of healthy and infected tomato plants. Similarly, Atallah et al. (2022) reported that plants treated with *A. niger* and *A. japonicus* showed the highest survival rates compared to untreated plants infected with the white mold disease of beans (*S. sclerotiorum*). The results suggest that this bio-control can be used as an ideal tool to combat the disease successfully.

### 4.3. Microscopic study of *A. niger* and *F. oxysporum* f. sp. *psidii* interaction

From the microscopic study, it was elucidated that *A. niger* has a bio-control mechanism that involves attaching, coiling, and lysing the pathogen with hydrolytic enzymes or secondary metabolites. The majority of bio-control fungi operate similarly, which causes the pathogen to die (Mukherjee et al., 2012; Duarte-Leal et al., 2018). Our findings support earlier reports where bio-control agents produced organic acids and extracellular enzymes that inhibited hyphal growth. Cell wall degrading enzymes include chitinase, β-1,3-glucanases, β-1,6-glucanases, α-1,3-glucanases, and proteases, which act on pathogens and lead to death (Patibanda and Sen, 2005; González et al., 2012). From the aforementioned results, it is confirmed that the ability of bio-control is mainly dependent on the enzymatic activity and secondary metabolite production capacity that restrict the growth and establishment of pathogens.

### 4.4. Secondary metabolites profiling

It is known that *A. niger* produces a variety of bioactive secondary metabolites with antifungal, antibacterial, and toxic properties against a variety of phytopathogens, primarily soil-borne pathogens. In the present study, secondary metabolites profiling using GC–MS identified several chemical compounds that belong to organic compounds, such as fatty acids, esters, alcohols, and phenols, having antimicrobial action. The compounds found during interaction in the hexane extracts have been reported to demonstrate antimicrobial activities. Fatty acids and their esters (hexadecanoic acid, methyl ester, *cis-*9-hexadecenoic acid, hexadecanoic acid, 9 or 6-octadecenoic acid, and 10-octadecenoic acid methyl ester), undecane, dodecane, tetramethyl benzene, 2,6-dimethyl undecane, 2-methyl dodecane, dimethyl octane, and trimethyl benzene majorly found. These metabolites have already been reported to have antifungal activity (Agoramoorthy et al., 2007; Imad et al., 2015; Casuga et al., 2016; Naqvi et al., 2019). The majority of the VOCs generated were fatty acids and their esters, which inserted themselves into the lipid bilayers of fungal membranes and caused an uncontrolled release of intracellular proteins and electrolytes, which finally caused the cytoplasmatic disintegration of fungal cells (Avis and Bélanger, 2001). The same fatty acids were also examined by Liu et al. (2008) against important phytopathogenic fungi, including *Alternaria solani, Colletotrichum lagenarium, F. oxysporum* f. sp. *cucumerinum*, and *F. oxysporum* f. sp. *lycopersici*, and reduced spore germination and mycelial growth of pathogens were seen. From ethyl acetate extract, major compounds, *viz*., acetic acid ethyl ester, benzopyron-4-ol, 1,2,6-hexanetriol, 2-propenoic acid ethanediyl ester, 1-(3-ethyloxiranyl) ethenone, 6-acetyl-8-methoxy dimethyl chromene, and octadecanoic acid, were found during the interaction to have antifungal activity. Idan et al. (2017) identified around 15 major compounds of *A. niger* against *P. oryzae* (Rice blast) using GC–MS (oleic acid, *n-*hexadecanoic acid (palmitic acid), hexose, glycerol, stearic acid, tetradecanoic acid, dodecanoic acid, and 5-hydrxoymethylfurfural), most of which belong to fatty acids.

Fatty acids (oleic acid (9-octadecenoic acid), *n*-hexadecanoic acid, and stearic acids) were also found effective against *Fusarium solani* f. sp. *pisi* (Jha and Jalali, 2006)*, Fusarium solani, Meloidogyne incognita* (Jang et al., 2016), *Candida* spp., *Micrococcus luteus, Pseudomonas aeruginosa*, and *Bacillus subtilis* (Agoramoorthy et al., 2007; Stenz et al., 2008). Furanacetic acid derivative (4-hexyl-2,5-dihydro dioxo furan acetic acid), similar to other furancarboxylic acid derivatives, has antibacterial and antifungal potential (Chang et al., 2020). Compounds having acids, esters, pyran, and furan from *A. niger* exhibited antibacterial activity against *Pseudomonas aerogenosa, Escherichia coli, Proteus mirabilis, Staphylococcus aureus*, and *Klebsiella pneumonia* (Imad et al., 2015). The compound 1-(3-Ethyloxiranyl) ethenone isolated from *Cordia obliqua* displayed antimicrobial properties against different microorganisms (Raj and Kumar, 2018). The compound 1,2,6-hexanetriol (a replacement of glycerin) is a sugar-derived alcohol, having antifungal activity, and chromenes (a derivative of coumarin) found in our experiment have been reported to show broad biological inhibitory antimicrobial properties (Basanagouda et al., 2010; Sahoo and Paidesetty, 2017; Ashok et al., 2018). Benzopyran-containing compounds are related to polyketide synthesis and are formed by the fusion of the benzene ring with the heterocyclic pyran ring. These are core ring structures of flavonoids, isoflavonoids, and isocoumarins, which have strong antifungal activity (Santiago et al., 2014). Most of the antifungal compounds identified from the study were produced during the interaction of bio-control and pathogens. These findings will serve as a foundation for the successful management of diseases in agriculture by using VOCs from the bio-control agent *A. niger*.

## 5. Conclusion

In recent years, biological control has received increasing attention as a promising alternative to chemical control of plant pathogens. In the present study, *A. niger* was found to be a promising antagonist for the management of the guava wilt pathogen FOP tested under lab and greenhouse conditions. Our study also suggested that the application of *A. niger* before the establishment of the disease reduces the wilt incidence significantly. GC–MS analysis revealed the production of antifungal secondary metabolites such as acetic acid, ethyl ester, benzopyron-4-ol, 1,2,6-hexanetriol, 2-propenoic acid ethanediyl ester, 6-acetyl-8-methoxy dimethyl chromene, 4-hexyl-2,5-dihydro dioxo furan acetic acid, octadecanoic acids, and some esters and acids, which may be involved in the mycoparasitism by acting synergistically. Our findings collectively imply that *A. niger* and its antimicrobial substances have great potential to successfully manage guava wilt and other soil-borne diseases. Since BCAs perform differently at varied climatic conditions, there is a need to evaluate their efficacy at multiple locations to confirm their robustness and potentiality.

## Data availability statement

The datasets presented in this study can be found in online repositories. The names of the repository/repositories and accession number(s) can be found in the article/Supplementary material.

## Author contributions

RG, DK, and AK were involved in the conceptualization of the project, study design, critical inputs, and finalization of the manuscript. RG contributed to the lab work and statistical analysis and wrote the first draft. RG, DK, AN, GP, and AD finalized the outline and prepared schematics. RG, AD, RD, GP, NB, and NG helped with statistical data analysis and editing of the manuscript. RG, AK, and VR carried out GC–MS work and analysis. GC captured SEM images. All authors contributed to the article and approved the submitted version.
