# Supplementary material for: Metabolomic profiling and its association with the bio-efficacy of Aspergillus niger strain against Fusarium wilt of guava
Source: Front Microbiol. 2023 Apr 24;14:1142144. doi: 10.3389/fmicb.2023.1142144 (PMC10165087; doi:10.3389/fmicb.2023.1142144)
Supplement: Supplementary file 1 [file Data_Sheet_1.doc]

Supplementary Material

**“Metabolomic profiling and its association with bio-efficacy of *Aspergillus niger* strain against *Fusarium* wilt of guava”**

Gangaraj R^1^, Aditi Kundu^2^, Virendra Singh Rana^2^, Amrita Das^1^, Gautam Chawla^3^, Prakash G^1^, Rubin Debbarma^1^, Nagaraja A^4^, Naresh Kumar Bainsla^5^, Navin Chandra Gupta^6^ and Deeba Kamil^1*^

^1^Division of Plant Pathology, ICAR-Indian Agricultural Research Institute, New Delhi, India.

^2^Division of Agricultural Chemicals, ICAR-Indian Agricultural Research Institute, New Delhi, India.

^3^Division of Nematology, ICAR-Indian Agricultural Research Institute, New Delhi, India.

^4^Division of Fruits and Horticultural Technology, ICAR-Indian Agricultural Research Institute, New Delhi, India.

^5^Division of Genetics, ICAR-Indian Agricultural Research Institute, New Delhi, India.

^6^ICAR-National Institute for Plant Biotechnology, New Delhi, India.

*Corresponding author: Deeba Kamil

email id: [deebakamil@gmail.com](mailto:deebakamil@gmail.com)

# Supplementary Figures


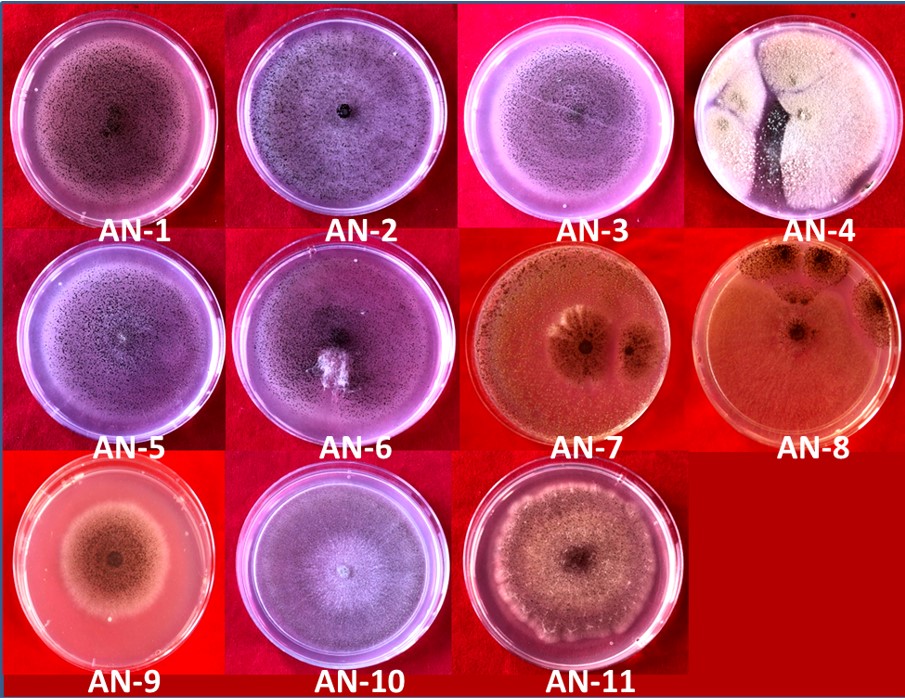


**Supplementary Figure 1:** Growth characteristics of different strains of *Aspergillus niger* on potato dextrose agar (PDA) after 7 days of incubation at 26± ºC.


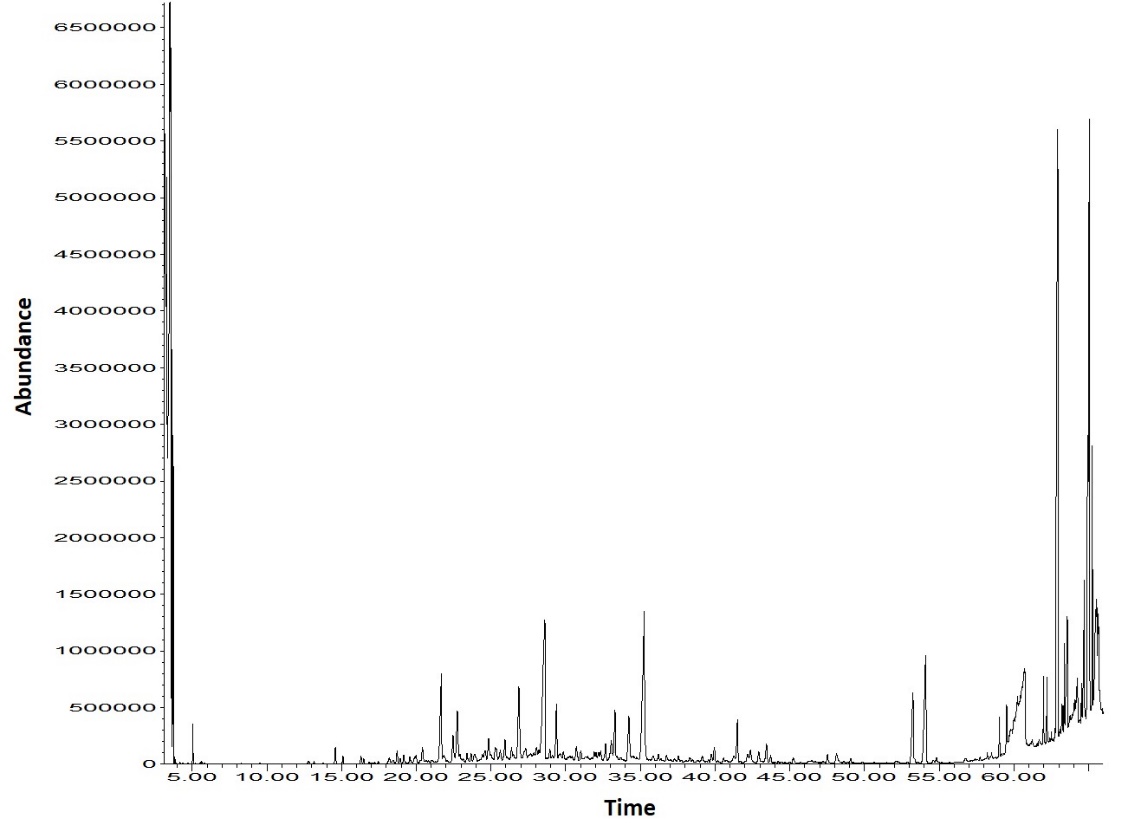


**Supplementary Figure 2:** GC-MS chromatogram representing the secondary metabolites from hexane fraction of *Fusarium oxysporum* f. sp. *psidii*.


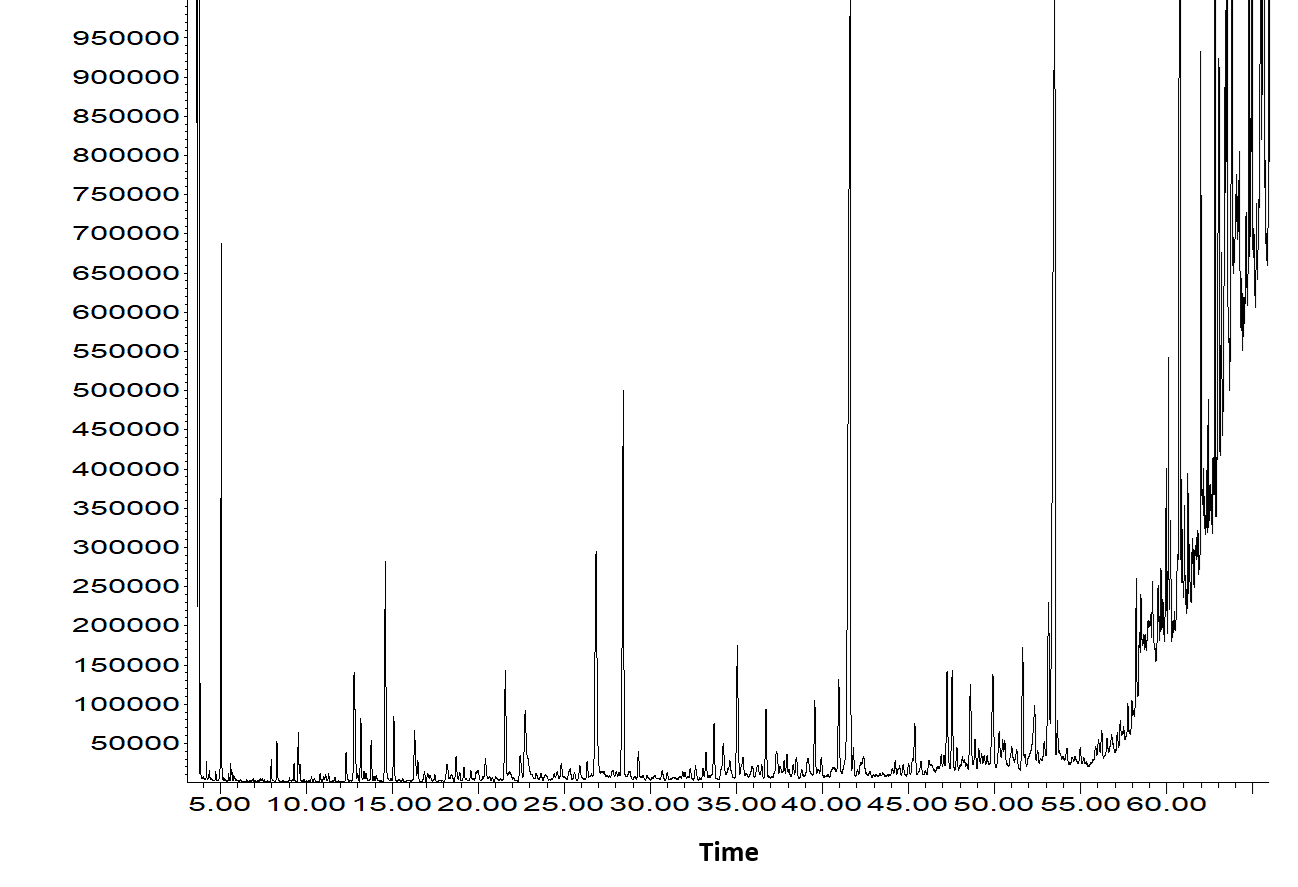


**Supplementary Figure 3:** GC-MS chromatogram representing the secondary metabolites from hexane fraction of *Aspergillus niger.*


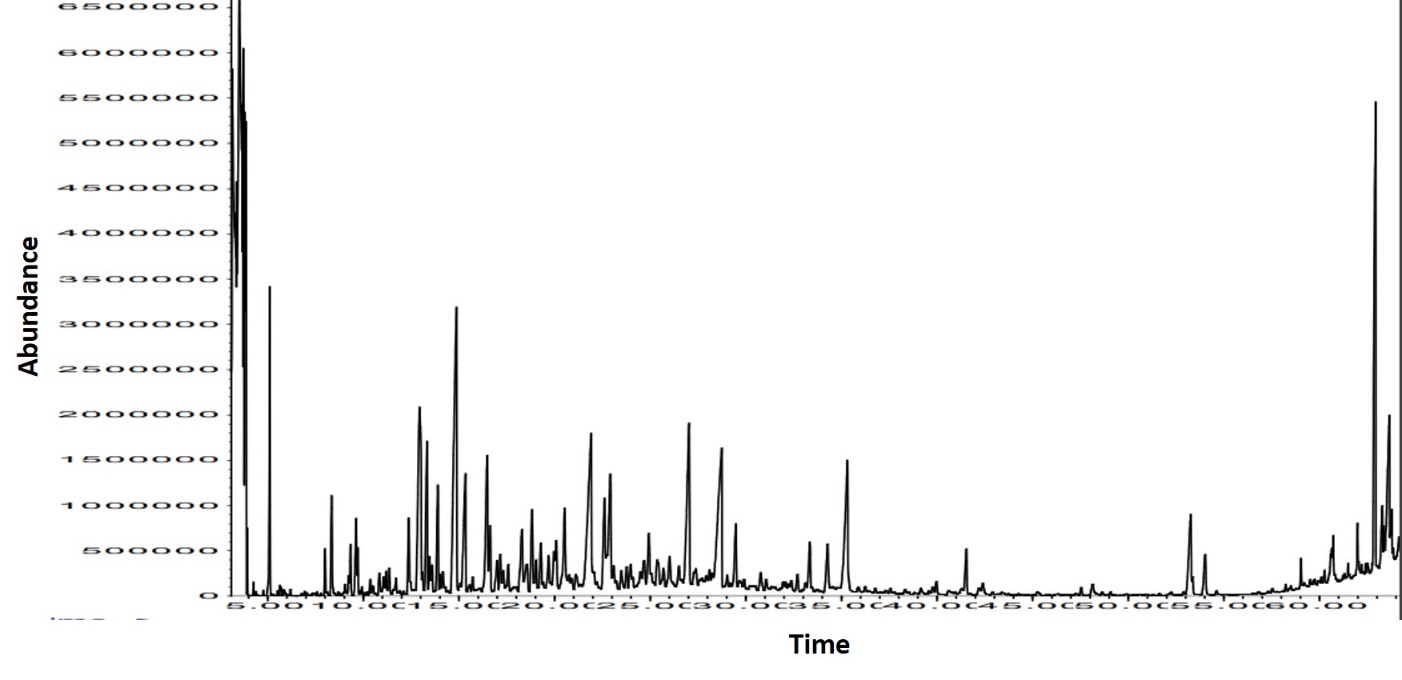


**Supplementary Figure 4:** GC-MS chromatogram representing the secondary metabolites from hexane fraction of *Fusarium oxysporum* f. sp. *psidii* and *A. niger* interaction*.*


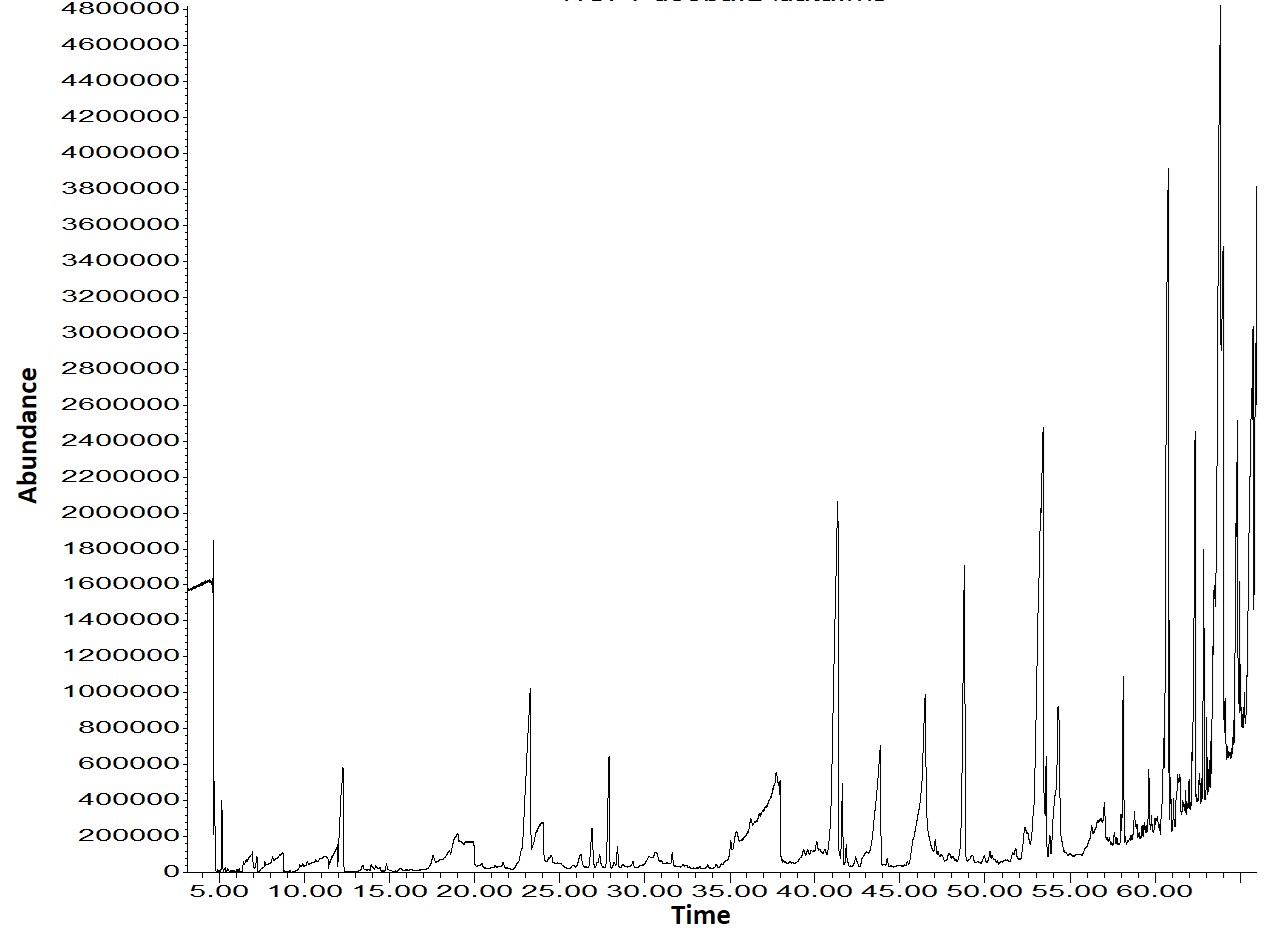


**Supplementary Figure 5.** GC-MS chromatogram representing the secondary metabolites from ethyl acetate fraction of *Fusarium oxysporum* f. sp. *psidii.*


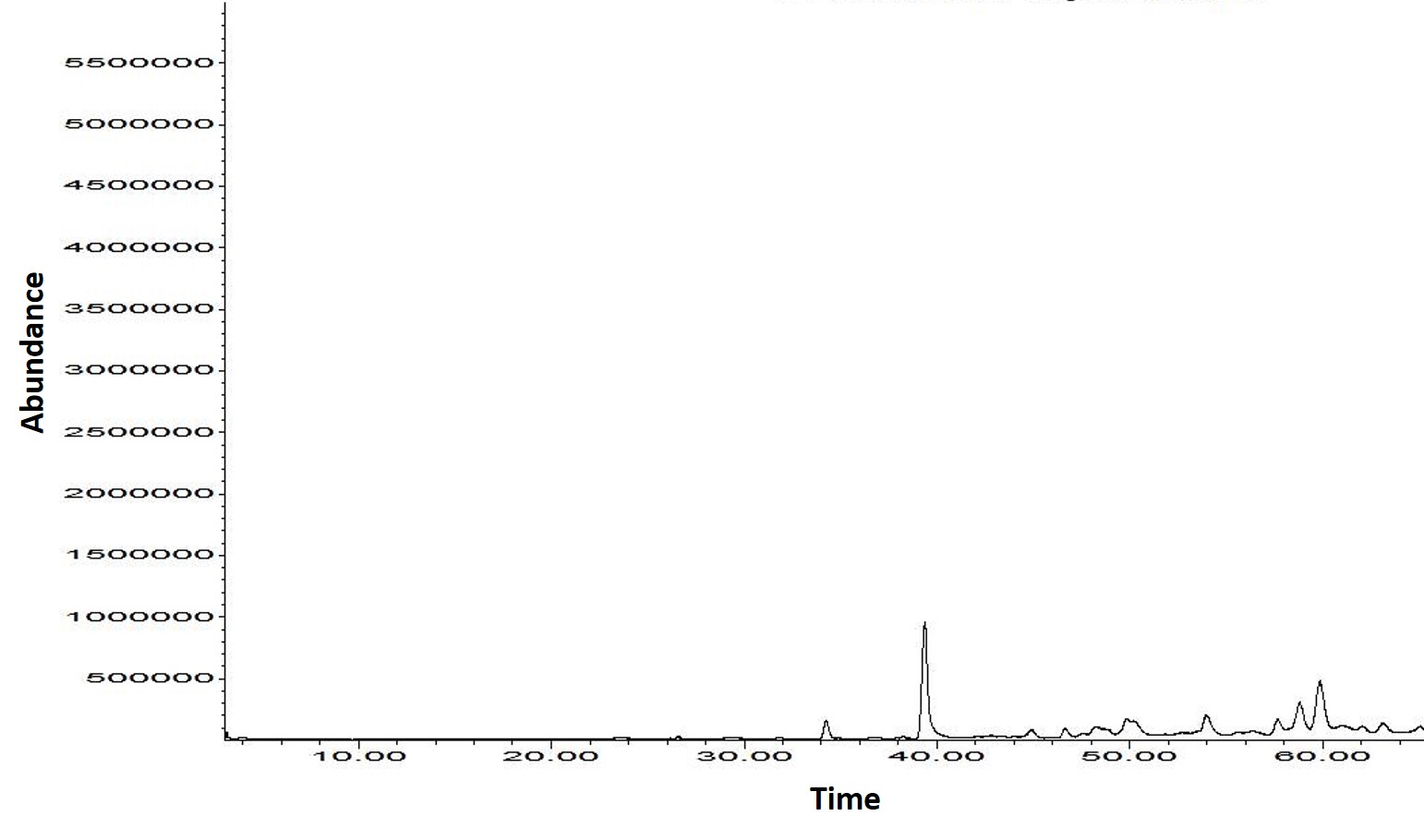


**Supplementary Figure 6.** GC-MS chromatogram representing the secondary metabolites from ethyl acetate fraction of *Aspergillus niger.*


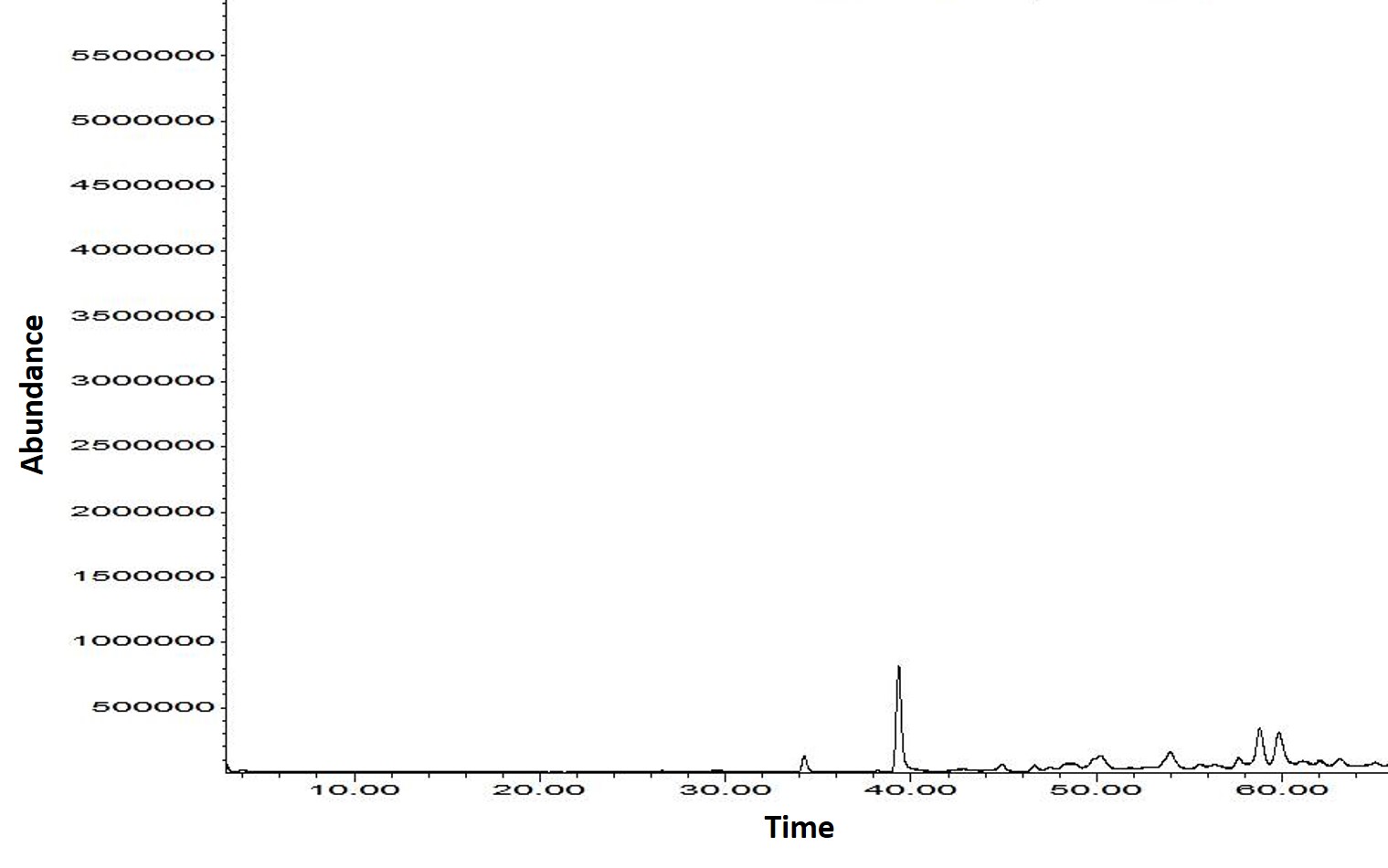


**Supplementary Figure 7.** GC-MS chromatogram representing the secondary metabolites from ethyl acetate fraction of *Fusarium oxysporum* f. sp. *psidii* and *A. niger* interaction.
